# Blackcap Optimization Algorithm (BCOA): A Novel Metaheuristic Algorithm for Global and Engineering Optimization Problems

**DOI:** 10.3390/biomimetics11060419

**Published:** 2026-06-13

**Authors:** Ali Asghari, Mohammadhossein Mohammadi

**Affiliations:** Department of Computer Engineering, Shafagh Institute of Higher Education, Tonekabon 4683165363, Iran; mohammadhossein.mohammadi@shafagh.ac.ir

**Keywords:** metaheuristic algorithms, Blackcap Optimization Algorithm (BCOA), engineering optimization problems, exploration and exploitation

## Abstract

Metaheuristic algorithms are widely used to find optimal or near-optimal solutions for complex problems by taking inspiration from natural behaviors and processes. Although many different methods have been developed, a common problem in many of them is maintaining a good balance between exploration and exploitation and avoiding local optima. To deal with this issue, this paper proposes a new method called the Blackcap Optimization Algorithm (BCOA), which is inspired by the navigation and migration behavior of Blackcap birds. Instead of using complicated distance calculations, the proposed method is based on angular movement vectors. The movement of each search agent is controlled by an angle-based mathematical model that combines the global best angle, a successful neighboring angle, and an adaptive exponential disturbance factor. In addition, the algorithm uses a quasi-genetic path transition mechanism to combine successful parent paths together, along with a territorial competition stage. This structure helps reduce computational cost and improves the balance between exploration and exploitation. The performance of the proposed algorithm is tested on 32 benchmark functions and seven engineering and network optimization problems. The simulation results show that BCOA has a good ability to avoid local optima and can achieve acceptable convergence speed and cost reduction compared to several existing methods.

## 1. Introduction

Optimization is one of the fundamental topics in many scientific and engineering areas. In a wide range of applications, such as engineering design, energy system planning, machine learning, scheduling, data processing, and resource management, the goal is to find the best or a near-optimal solution among a set of possible options. However, many real-world problems have characteristics such as nonlinearity, multimodality, high dimensionality, and complex constraints, which make classical optimization methods difficult to apply. Deterministic and gradient-based approaches [1] usually require detailed information about the structure of the objective function, and they often get trapped in local optima or fail when the function is nondifferentiable. These limitations encouraged researchers to develop more flexible search methods that do not rely heavily on problem structure. In response to this need, a family of intelligent search methods known as metaheuristic algorithms [2] emerged. These algorithms are usually inspired by natural phenomena, collective behavior of living organisms, or physical processes, and they try to explore the search space efficiently through interactions among search agents. One of the earliest and most influential examples is the Genetic Algorithm [3] introduced by Holland in the 1970s, which uses concepts such as selection, mutation, and crossover for exploring the solution space. Later, other approaches such as Simulated Annealing [4], inspired by thermodynamic processes, and Tabu Search [5], designed to escape local optima, were proposed. These methods showed that probabilistic mechanisms and search memory can significantly improve the ability to solve complex optimization problems.

In the following decades, with the progress in swarm intelligence research, a new generation of metaheuristic algorithms inspired by the collective behavior of living species appeared. Well-known examples include Particle Swarm Optimization (PSO) [6], inspired by the flocking behavior of birds and fish; Ant Colony Optimization (ACO) [7], inspired by food-seeking patterns in ants; and many other algorithms motivated by the behavior of bees [8], wolves [9], whales [10], and various animal species introduced in the last two decades. These algorithms typically rely on a group of search agents that move toward promising regions by sharing information and updating their positions. The success of these methods made metaheuristic optimization one of the most active research fields in recent years.

Despite these developments, a closer look at the literature shows that many existing algorithms still face important challenges. One major challenge is achieving a proper balance between exploration and exploitation [11]. Some methods are good at global search in the early stages but fail to converge stably later on; others quickly focus on narrow regions and risk premature convergence [12]. Furthermore, many algorithms require several control parameters, and tuning them depends on user experience or extensive experimentation. These issues indicate that there is still considerable potential for designing new search algorithms with dynamic, simple, and effective behavior. One promising direction for designing new metaheuristics is to take inspiration from natural behavioral patterns where adaptive mechanisms, collective interactions, and information exchange occur naturally. Migratory behavior in many animal species is an example of such complex systems, where collective decision-making, learning from experience, and environmental adaptation happen together. Among these species, the blackcap bird (*Sylvia atricapilla*) [13] has attracted attention due to its flexible migration routes. The migration path of this bird is not a fixed route but a result of combining several factors, including genetic tendencies, individual experience, environmental conditions, and interactions with other members of the population. One important feature is the inheritance of migration direction from parents to offspring, where young birds often choose a direction between the routes of their parents and gradually refine it during migration.

In this research, inspired by this natural behavior, a new metaheuristic called the Blackcap Optimization Algorithm (BCOA) is introduced. In this algorithm, each individual in the population represents a migratory bird, and its position indicates a candidate solution in the search space. Unlike many population-based algorithms, where individuals directly move toward better positions, in BCOA, the search dynamics are based on the movement direction or migration angle. In this way, the search process is guided by gradual adjustments in direction, influenced by the best individual and successful neighbors. This angle-based design allows a natural balance between wide exploration and gradual convergence toward promising areas. The proposed model includes several key behaviors observed in blackcap migration. These include the inheritance of movement direction between generations, gradual path correction using collective experience, and territorial interactions among individuals in occupied areas. Combining these behaviors helps the search population explore the problem space dynamically while converging toward high-quality regions.

The main contributions of this research can be summarized as follows:

- Introducing an angle-based search framework that models the movement of search agents through migration direction.
- Developing a directional inheritance mechanism across generations using weighted parent fitness.
- Designing an adaptive angle-correction process based on collective experience and interaction with successful neighbors.
- Modeling territorial competition among individuals to improve spatial diversity and prevent premature convergence.
- Presenting a consistent mathematical model that allows analysis of movement behavior in a multi-dimensional space.

The rest of the paper is organized as follows. Section 2 reviews related research and existing metaheuristic algorithms. Section 3 explains the mathematical model and the operational steps of the proposed BCOA. Section 4 presents the experimental evaluation and comparative analysis. Finally, Section 5 concludes the study and offers directions for future work.

## 2. Literature Review

Metaheuristic algorithms have become effective stochastic optimization methods for solving complex, nonlinear, and high-dimensional problems, especially when finding the exact global optimum is computationally difficult for traditional deterministic methods. By imitating different natural and social phenomena, these algorithms try to maintain a balance between exploration of the global search space and exploitation of promising local regions in order to reach near-optimal solutions within a reasonable computational time. In optimization studies, based on their main source of inspiration, metaheuristic algorithms are generally divided into four categories: Evolutionary algorithms, inspired by biological evolution and genetic principles; Swarm Intelligence algorithms, inspired by the collective behavior of animals and living organisms; Physics-based algorithms, derived from physical laws, forces, and dynamic processes; and Human-based algorithms, which simulate human social interactions, learning, and decision-making behavior. The following sections present a review of important and recently developed algorithms in each of these four categories.

Evolutionary-based algorithms, as a key class of search and optimization methods, are directly inspired by biological evolution principles such as selection and genetic mutation, and they have a strong ability to escape local optima in complex spaces. Recent research in this field mainly focuses on developing adaptive structures and improving efficiency when facing hard constraints. In [14], the Genetic Algorithm (GA) is introduced as the fundamental core and the most well-known evolutionary method, which established the main framework for exploration in nonlinear search spaces by accurately simulating chromosomal crossover and mutation mechanisms. The research presented in [15] describes the Heterogeneous Alternating Evolutionary Algorithm (HAEA) framework. This method uses advanced information indices to dynamically integrate several evolutionary sub-algorithms, showing high adaptability in different stages of the optimization process. In [16], a Gaussian model-based multi-objective algorithm (KG-DMOEA) is proposed that optimizes decision variables and handles dynamic and time-varying environments with high accuracy by focusing specifically on identifying and exploring knee points on the Pareto front. The reviewed paper in [17] discusses a multi-population algorithm based on constraint grouping (CGMEA). By creating independent populations based on problem constraints and using an innovative auxiliary competition mechanism, it creates a much better balance between local and global search in constrained problems. In [18], an extended human evolutionary algorithm (HEST-HEO) is presented. To significantly improve convergence performance in feature selection problems, it uses a combination of the Halton mathematical sequence and the targeted application of t-distribution perturbation to avoid falling into local optima traps. Researchers in [19] developed a multiple probability distribution-based framework (EA-mPD). This structure is designed to deal with the uncertainty challenge and intelligently clusters populations to use local models for more accurate fitness estimation. In [20], a decomposition-based multi-objective algorithm (MOEA/D-NRD) is considered. By introducing the novel concept of neighborhood region dominance and continuous comparison with local ideal points, it improves the diversity of solution distribution in complex multi-objective problems. The study in [21] introduces the Love Evolution Algorithm (LEA) with an interdisciplinary psychological approach. This framework is developed based on the stimulus-value-role theory and simulates the optimization process in three sequential phases that are completely matched with the evolution stages of human relationships. In [22], a dynamic multi-objective algorithm (AB-DMOEA) is described. Relying on an adaptive boosting mechanism and prediction strategies, it can track time-varying Pareto fronts with much more speed and stability compared to traditional methods. The advancements presented in [23] revolve around a competitive-cooperative co-evolutionary algorithm (MSCOEA). By continuously monitoring the diversity of sub-populations through an adaptive random competition system, it effectively prevents premature convergence and maintains global exploration ability. In [24], a customized model-based evolutionary algorithm is presented that is specifically designed to solve complex path optimization and precise scheduling problems in healthcare distribution networks, considering hard time constraints. In [25], the Chaos-based Phasmatodea Population Evolution (CPPE) algorithm is introduced. By replacing the random initialization process with complex mathematical maps, it increases the initial dynamics and diversity of the population, thereby strongly improving the search power. Finally, in [26], the Trees Social Relations Optimization Algorithm (TSR) was introduced, inspired by the hierarchical life and collective consciousness of trees in a forest. This swarm intelligence algorithm uses a mechanism of parallel and synchronized sub-jungles to facilitate communication among trees and protect young seedlings. The evaluations showed that this cooperative structure improves the accuracy of solutions and reduces the convergence time for solving a wide range of continuous and discrete optimization problems compared to similar algorithms. Other algorithms in this category include Artificial Gorilla Troops Optimizer (AGTO) [27], African Vultures Optimization Algorithm (AVOA) [28], Beluga Whale Optimization (BWO) [29], multi-level prediction and elite individual mutation strategy (MPEIS) [30], Walrus Optimization Algorithm (WaOA) [31], Snow Leopard Optimization Algorithm (SLOA) [32], Giant Trevally Optimizer (GTO) [33], and Fennec Fox Optimization (FFO) [34].

Swarm-based algorithms, inspired by nature, have been developed to solve complex optimization problems. The foundation of many of these methods is the Particle Swarm Optimization (PSO) algorithm. In [35], this algorithm is introduced by modeling the coordinated search behavior and social information sharing of a group of particles moving towards the best individual and global positions. In [36], to overcome the challenges of complex decision spaces in large-scale multi-objective problems, the adaptive competitive swarm optimizer (AMRCSO) is developed. This method prevents premature convergence and balances exploration and exploitation by using parallel multi-directional exploration and an adaptive competition mechanism. Also, in [37], a migration model based on a multi-swarm system (MMPSO) is presented, inspired by natural migration phenomena. This model uses multi-vertex convergent search and an automatic transfer system to maintain population diversity, which effectively helps to solve complex multimodal problems and escape from local optima. In the field of multi-objective problems, in [38], the grid-based version of the cat swarm optimization (GMOCSO) is proposed. This algorithm improves diversity preservation and overall convergence speed by replacing the traditional roulette wheel method with a greedy approach and integrating a dual archive strategy to manage Pareto optimal solutions.

Dealing with high-dimensional data, such as feature selection, in [39], the dynamic multi-swarm whale optimization algorithm (EMSWOA) is introduced. This approach corrects search errors caused by invalid changes using the center of mass distance criterion and an elite adjustment mechanism, which improves the stability of the selection process. In order to prevent the decrease in search efficiency in high iterations, in [40], the improved sand cat swarm optimization (ISCSO) is presented. This method improves the global search capability and local exploitation by combining low-frequency noise search, spiral shrinking movement strategies, and opposition-based learning. In [41], the basic Whale Optimization Algorithm (WOA) is formulated by mathematically simulating the bubble-net hunting strategy of humpback whales. This algorithm utilizes a spiral encircling mechanism and random search agents to effectively locate prey in the search space. Furthermore, in [42], the Harris Hawks Optimization (HHO) algorithm is developed. This method models the cooperative behavior and surprise attacks of these birds from different directions, presenting dynamic chasing patterns that adapt to the escaping energy of the prey.

In marine environments, in [43], the Marine Predators Algorithm (MPA) is proposed based on the Brownian and Lévy motion strategies of ocean predators. It also considers the optimal encounter rate policy between predator and prey, providing a flexible structure for both exploration and exploitation phases. For an accurate estimation of the Pareto front, in [44], the multi-objective version of the African vulture optimization algorithm (MOAVOA) is introduced. This algorithm is developed by combining advanced mechanisms such as archiving, gridding, and leader selection, which enhance the distribution and quality of the extracted solutions. The social intelligence of mammals has also received attention, such that in [45], the Chimp Optimization Algorithm (ChOA) is proposed. This algorithm divides the population into four distinct roles—attacker, barrier, chaser, and driver—to accurately simulate the stages of group hunting and improve the search space exploration. In [46], the Artificial Bee Colony (ABC) algorithm is developed by simulating the intelligent foraging behavior of honey bees. This algorithm divides bees into employed, onlooker, and scout groups to continuously improve solutions through a dynamic neighborhood search mechanism. In [47], the Butterfly Optimization Algorithm (BOA) is introduced by modeling the food search and mating behavior of butterflies. This method is based on fragrance concentration analysis, where butterflies move toward the individual emitting the strongest scent, facilitating convergence to the global optimum. Finally, in [48], the Farmer Ants Optimization Algorithm (FAOA) was introduced, inspired by the life of these ants in cultivating mushrooms. This algorithm models the social behavior of the ants in protecting and nourishing fungi as its search mechanism. The experimental results on classical and engineering problems showed that this algorithm provides acceptable solutions for discrete optimization problems. Other algorithms in this category include Dung Beetle Optimizer (DBO) [49], Osprey Optimization Algorithm (OOA) [50], Coati Optimization Algorithm (COA) [51], Crested Porcupine Optimizer (CPO) [52], Northern Goshawk Optimization (NGO) [53], Nutcracker Optimization Algorithm (NOA) [54], and Honey Badger Algorithm (HBA) [55].

Physics-based optimization algorithms are developed inspired by nature’s laws and physical phenomena to solve complex problems. The foundation of many of these methods is the Simulated Annealing (SA) algorithm; in [56], this algorithm is introduced by modeling the annealing process and the gradual reduction in energy E to escape local optima. In [57], the Gravitational Search Algorithm (GSA) is developed, where solutions are considered as physical masses and move towards heavier masses through Newton’s law of gravity and the exchange of force F. Also, in [58], the Big Bang-Big Crunch (BB-BC) algorithm is presented, inspired by the universe evolution theory, which includes two phases of generating random solutions in the bang stage and focusing around the center of mass in the crunch stage. To investigate particle interaction, in [59], the Charged System Search (CSS) is proposed, which models the attraction and repulsion forces between particles based on electrostatic and Newtonian mechanics laws. In [60], Central Force Optimization (CFO) is introduced as a deterministic approach that simulates the movement of probes under the influence of gravitational forces in a three-dimensional space.

With the expansion of physical concepts in optimization, in [61], the Black Hole Algorithm (BHA) is proposed, where the best solution acts as a black hole and pulls other stars towards itself and replaces them if they cross the event horizon. In [62], the Multi-Verse Optimizer (MVO) is developed using cosmology concepts like white holes and wormholes to create a proper balance between exploration and exploitation. In the thermodynamics field, in [63], Thermal Exchange Optimization (TEO) is introduced based on Newton’s law of cooling, which models heat exchange between objects until reaching an equilibrium state. To maintain stability in dynamic systems, in [64], the Equilibrium Optimizer (EO) is presented, inspired by mass balance models in physics, which uses a base state to guide the particles. In [65], the Archimedes Optimization Algorithm (AOA) is formulated relying on the buoyancy law, where objects move based on their density and volume. Furthermore, in [66], Henry Gas Solubility Optimization (HGSO) is developed by mimicking the solubility behavior of gases in liquids under different pressures.

At the subatomic level, in [67], the Atomic Orbital Search (AOS) algorithm is introduced using quantum mechanics principles and modeling the behavior of electrons in layers around the nucleus. To analyze electrical systems, in [68], Transient Search Optimization (TSO) is developed, inspired by the behavior of circuits including resistors, inductors, and capacitors. In [69], the Kepler Optimization Algorithm (KOA) is proposed, utilizing Kepler’s laws of planetary motion, which updates the position of solutions based on elliptical orbits. In [70], the Fick’s Law Algorithm (FLA) is modeled based on the laws of diffusion and mass transfer from high- to low-concentration areas. Also, in [71], Electromagnetic Field Optimization (EFO) is presented, inspired by the polarity of electromagnets and their interaction. In the optics area, in [72], the Light Spectrum Optimizer (LSO) is developed based on light scattering and refraction phenomena, and in [73], Water Evaporation Optimization (WEO) simulates the physics of molecule evaporation. In [74], Ray Optimization (RO) is formulated based on Snell’s law of refraction and the movement of light rays. In [75], Colliding Bodies Optimization (CBO) is introduced using the laws of momentum and energy conservation in elastic collisions to maintain population diversity during the search. Finally, in [76], the Water Optimization Algorithm (WAO) was introduced, inspired by the physical and chemical properties of water molecules. This algorithm creates a new search mechanism using concepts such as the movement, evaporation, and bonding of molecules to find optimal points. The evaluation results on standard functions and practical problems showed that this method provides acceptable solutions in terms of accuracy and execution time compared to similar algorithms.

Human-based algorithms are designed by taking inspiration from behavioral patterns, social interactions, and political or educational structures in human societies. By simulating collective decision-making and learning mechanisms, this category of algorithms provides innovative approaches for solving complex optimization problems. In [77], the Cultural Algorithm (CA) is introduced by modeling cultural evolution and knowledge transfer in two separate spaces: population space (micro-evolution) and belief space (macro-evolution). In this method, the experiences of top individuals are saved in the belief space and passed to the next generations. Its main achievement is a significant improvement in convergence in dynamic search spaces. In [78], the Imperialist Competitive Algorithm (ICA) is developed by simulating the competition among empires. This method includes the phases of moving colonies toward empires (assimilation) and sudden revolutions, which have provided high speed and accuracy in solving combinatorial and NP-Hard optimization problems. Also, in [79], the Teaching-Learning-Based Optimization (TLBO) algorithm is presented, inspired by a classroom environment in two consecutive phases: teacher (knowledge transfer from the best individual to others) and learner (interaction of individuals with each other). Because it completely removes user-tuned parameters, it has shown high stability in continuous functions. In [80], the Brain Storm Optimization (BSO) algorithm models the idea generation process in human groups through clustering ideas and creating new ones based on combining clusters. Its achievement is keeping population diversity and successfully escaping local optima in complex multimodal problems. In [81], the Exchange Market Algorithm (EMA) is developed by imitating the behavior of shareholders in financial markets under oscillation and stable conditions. By dividing shareholders into two groups of risk-taking and risk-avoiding, this algorithm has been very successful in solving economic dispatch problems and complex financial modeling.

With the expansion of social and political interactive models, in [82], the Election Algorithm (EA) simulates the behavior of political parties and voters in election campaigns. Using mechanisms of positive advertisement, negative advertisement, and coalition formation, this method has achieved high accuracy in resource allocation problems. In [83], the Poor and Rich Optimization (PRO) algorithm is formed by investigating the class gap and wealth distribution in society. In this mechanism, rich individuals try to keep their position, and poor individuals try to reduce the class gap by copying the richest ones. This approach has created a very good balance between exploration and exploitation phases. In [84], the Political Optimizer (PO) is proposed by modeling multi-party systems and the power transfer process in governments. This method includes the phases of party formation, party switching, and parliamentary elections, and its achievement is fast global convergence in structural engineering design problems. In [85], the Gaining-Sharing Knowledge-based Algorithm (GSK) models the stages of human learning over a lifetime. This algorithm has two main phases: gaining knowledge in youth (from close people) and sharing knowledge in adulthood (from wider networks). It has shown superior performance in high-dimensional optimization spaces. Also, in [86], Social Network Search (SNS) models the interactions of users in virtual networks. This method uses four main operators (imitation, conversation, innovation, and browsing) to update the position of users, which has reached very competitive results in solving complex problems.

In [87], the War Strategy Optimization (WSO) algorithm is presented by modeling the military tactics of soldiers on the battlefield. This algorithm simulates the movement of troops based on offensive and defensive strategies, which has shown great robustness and stability in controlling nonlinear systems in 2025 studies. In [88], the Driving Training-Based Optimization (DTBO) algorithm simulates the driving learning process in three phases: training by an instructor, imitating expert drivers, and personal practice. In novel applications of 2025, this method could tune the hyperparameters of deep learning networks with high accuracy. In [89], the Language Education Optimization (LEO) algorithm is developed, inspired by foreign language learning methods. This method includes listening, speaking, reading, and writing phases to improve vocabulary (optimal position). The research literature in 2025 and 2026 has confirmed its notable success in scheduling problems. Finally, in [90], the Teamwork Optimization Algorithm (TOA) simulates group activities to reach a common goal. This method works by dividing tasks, receiving guidance from the supervisor, and individually executing tasks. Its developed versions in 2026 have achieved great results in renewable energy management. Other algorithms in this category include Human Learning Optimization (HLO) [91], Group Teaching Optimization Algorithm (GTOA) [92], Student Psychology-Based Optimization (SPBO) [93], Soccer League Competition (SLC) [94], Gold Rush Optimizer (GRO) [95], and Chef-Based Optimization Algorithm (CBOA) [96].

Despite the large number of studies on metaheuristic optimization algorithms, several important gaps still exist in the literature. As observed in evolutionary, swarm intelligence, physics-based, and human-inspired algorithms, many methods have been proposed to improve convergence speed, preserve population diversity, and avoid local optima. However, in most existing algorithms, the movement of search agents is mainly modeled through direct position updates in the search space, while the role of movement direction is usually treated implicitly or considered only as a secondary factor. This issue may cause unstable search paths, sudden movements in the search space, and limited control over the exploration process, especially in complex high-dimensional problems. In addition, diversity mechanisms in many algorithms are commonly created through random perturbations or fixed parameters, while structured inheritance mechanisms that can gradually guide the behavior of new individuals have received less attention. Furthermore, interactions among agents are often simplified and do not fully reflect realistic competitive behaviors that could dynamically balance exploration and exploitation.

**Figure 1:**
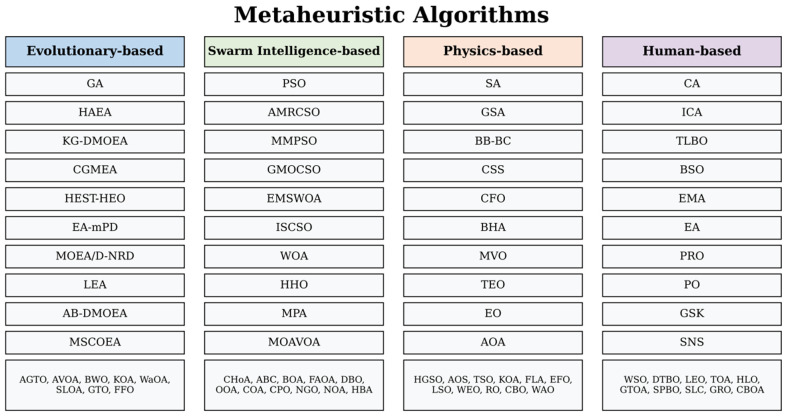
The classification of metaheuristic algorithms. Each category is shown in a separate color.

To overcome these limitations, the proposed Blackcap Optimization Algorithm (BCOA) introduces a direction-based search framework inspired by the migration behavior of blackcap birds. In this approach, the search process is mainly guided by the migration angle of individuals rather than only their positions, which allows smoother and more controlled search trajectories in the problem space. In addition, a reproduction mechanism based on the inheritance of migration direction from parent birds creates guided diversity in the population and enables offspring to explore different regions of the search space in a structured way. Moreover, the territorial competition mechanism models the interaction between resident and migrant birds, helping regulate population distribution and reduce premature convergence. By combining direction-based movement, inheritance of migration tendencies, and territorial competition, the BCOA framework presents a more realistic behavioral model that can improve the balance between exploration and exploitation and enhance search performance in complex optimization problems. Figure 1 shows the classification of metaheuristic algorithms.

## 3. The Blackcap Optimization Algorithm (BCOA)

In this section, the source of inspiration for the proposed algorithm is explained, followed by a detailed description of the mathematical model and its operators.

### 3.1. Inspiration

The blackcap (*Sylvia atricapilla*) is a well-known European songbird that has attracted the attention of biologists because of its diverse and flexible migratory behavior [97]. This species breeds in large parts of Europe and in some regions of Western Asia. When the cold season begins, many populations migrate toward warmer areas in southern Europe, North Africa, or other milder regions. Migration in this species is not a simple or linear process. It results from the interaction of several behavioral and biological factors, including inherited orientation tendencies, individual experience, environmental conditions, and interactions with other individuals in the population. The movement direction of blackcaps has a partly inherited component. Studies have shown that many young individuals have a natural tendency to move at a particular angle relative to their birthplace. This angle usually falls within a range that can be seen as an average of the migration directions of the parents or the source population. Experimental studies on blackcaps have shown that migratory orientation has a genetic basis. For example, Helbig [98] reported that hybrid offspring of populations with different migration directions tend to orient in an intermediate direction, which indicates inherited orientation tendencies.

However, this initial tendency is not completely fixed. During migration, it may change due to environmental conditions, food availability, or the bird’s own experience. As a result, the migration route in this species often emerges as a combination of an initial orientation and a gradual correction of the path over time. During migration, blackcaps usually travel in stages. They land at different points along the route to rest and to obtain food resources needed for the next part of the journey. These temporary stops allow the bird to evaluate the local environmental conditions and, if the resources are suitable, to remain in that area for some time. In some cases, these stops are short and temporary, and the bird continues its journey after recovering energy. In other cases, the same location may become a longer-term residence.

One serious situation that often occurs during these temporary stops is the encounter between migrants and resident individuals of the same species in the destination region. Field observations also indicate that territorial interactions among blackcaps can influence settlement and habitat selection, especially when individuals compete for limited food resources or resting areas [99]. Resident birds usually have already established specific territories for feeding and resting, and they often react to the arrival of newcomers. When a migratory bird enters such an area, a form of territorial interaction usually occurs between the migrant and the resident bird. If the competitive pressure is low or food resources are abundant, the resident bird may tolerate the presence of the migrant, allowing it to remain in the area and use the available resources. In such conditions, the migratory bird may effectively settle in that region and stop its movement. In contrast, if the resident bird has a well-established territory or if resources are limited, the interaction between the two individuals can become more intense. Migratory birds often arrive after a long flight with limited energy reserves. Because of this, they may have lower competitive ability compared with local birds. As a result, they may lose the competition and be forced to leave the area. Such rejection usually causes the migratory bird to move to nearby regions and search for a more suitable place to stop. Sometimes this relocation happens several times until the bird eventually finds a location where competition is lower or resources are sufficient. This process of encounter, acceptance, or rejection leads migrants to distribute themselves around the territories of resident populations and to explore several nearby areas. Consequently, the actual migration path can become wider and more complex than the initial route that the bird had at the beginning of its journey.

Along with these relatively common behaviors, part of the population often shows patterns that differ from the dominant migration trend. Some birds, especially younger individuals, may move without strictly following the typical migration routes of the population, or they may change their path unexpectedly during the journey. These deviations may occur because of navigation errors, individual behavioral differences, or responses to specific environmental conditions. Although many of these alternative movements may not be successful, in some cases, they allow birds to reach new regions that were not previously included in the common migration routes.

**Figure 2:**
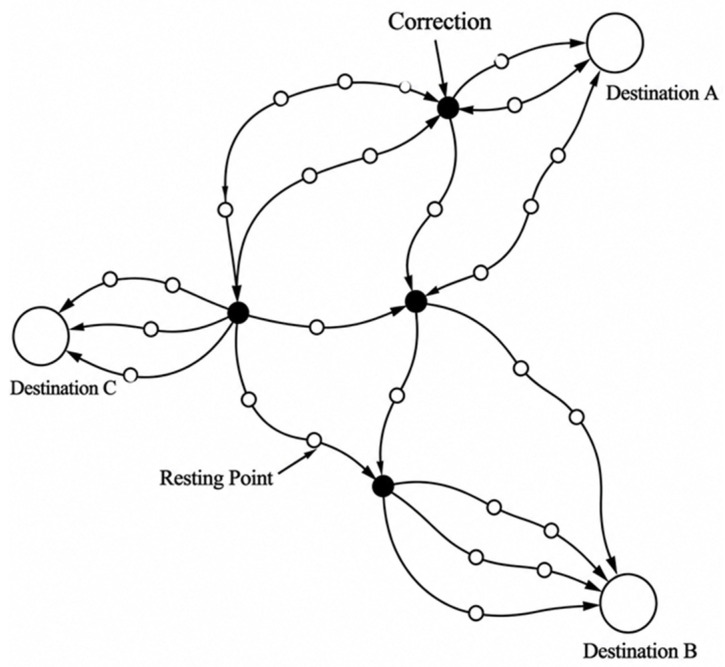
Migration Patterns of European Blackcap. Small hollow circles indicate resting points, filled circles indicate change of direction, large circles indicate migration destinations, and arrows indicate the direction of movement.

The existence of such behavioral diversity within the population, together with competitive interactions with resident birds and gradual correction of migration paths, keeps the distribution patterns of blackcap birds dynamic and flexible. This combination of behaviors—including inherited initial orientation, path correction during movement, evaluation stops along the route, competition with resident populations, and the appearance of unconventional behavior in some individuals—creates a complex framework of decision-making and behavioral adaptation in this species. Studying these mechanisms can help improve the understanding of movement dynamics in migratory populations. Figure 2 shows the migration pattern of the European Blackcap [100].

### 3.2. BCOA Mathematical Model

In this study, a new metaheuristic algorithm called the Blackcap Optimization Algorithm (BCOA) is proposed. This algorithm is inspired by the migration pattern of the blackcap bird, a species that adjusts its migration routes based on a combination of genetic tendencies, collective experience, and environmental interactions. One important feature of this bird is the inheritance of migration direction from parents to the next generation. Young birds usually select a direction that lies between the migration routes of their parents. In addition, the migration path is gradually corrected during the journey in response to environmental conditions and interactions with other birds. In the proposed algorithm, each individual in the population represents a migratory bird, and its position denotes a candidate solution in the search space. Unlike many population-based algorithms, where individuals move directly toward better positions, in BCOA, the movement dynamics are modeled based on the migration direction (movement angle). As a result, the search process is driven by gradual changes in the movement direction of individuals in the problem space. This angle-based framework allows a more natural modeling of migratory behavior and helps to create a balance between exploration and exploitation.

In the following, the mathematical model of the algorithm is introduced step by step. The optimization problem is defined in a D-dimensional space. Each point in this space is a candidate solution:

(1) $$X=(x_{1},x_{2},\ldots ,x_{D})$$

where D is the number of decision variables. The algorithm includes N birds, and each bird represents one solution:

(2) $$X_{i}^{t}=(x_{i1}^{t},x_{i2}^{t},\ldots ,x_{iD}^{t})$$

where i=1,2,…,N is the bird index, t is the iteration number, and $X_{i}^{t}$ is the position of bird i in the search space.

After defining the basic movement mechanisms, some important biological behaviors observed in blackcap migration should also be included in the model. In the proposed algorithm, each bird is a search agent in the problem space, and the set of agents forms an initial population of size N. Each individual $X_{i}$ is a candidate solution in the search space, and its quality is evaluated by the objective or fitness function $f(X_{i})$. The algorithm runs for T iterations, and in each iteration, the position and movement direction of individuals are updated according to migration rules. In this framework, the best solution found in the whole population is denoted by $X_{g}$, which has the best fitness value among all individuals. One important biological behavior considered in the model is reproduction and inheritance of migration direction to offspring. In nature, each pair of birds can produce several chicks, each with its own orientation tendency, although this tendency is usually an average of the parents’ routes.

In each iteration, parent birds are selected from the current population using a fitness-based probabilistic selection strategy. Since the problem is formulated as a minimization task, individuals with lower objective function values represent better solutions. The selection probability of each individual is defined as

$$P_{i}=\frac{1/f(X_{i})}{\sum_{k=1}^{N}(1/f(X_{k}))}$$

This Equation ensures that individuals with better fitness have a higher probability of being selected as parents. Two distinct individuals are then selected as male (p) and female (m) parents. Self-pairing is not allowed.

In the proposed model, each pair $\left(p\backslash \mathrm{male},m\backslash \mathrm{female}\right),$ produces two offspring whose initial migration angles are similar but not identical. The migration angles of the first and second offspring are defined as Equations (3) and (4).

(3) $$\theta_{c_{1}}^{0}=\frac{w_{p}\theta_{p}+w_{m}\theta_{m}}{w_{p}+w_{m}}+\lambda_{1}(\theta_{p}-\theta_{m})+\varepsilon_{1}$$

(4) $$\theta_{c_{2}}^{0}=\frac{w_{p}\theta_{p}+w_{m}\theta_{m}}{w_{p}+w_{m}}-\lambda_{2}(\theta_{p}-\theta_{m})+\varepsilon_{2}$$

In these equations, $\theta_{p}$ and $\theta_{m}$ are the migration angles of the male and female parents, and $w_{p}$ and $w_{m}$ are weights proportional to the fitness of each parent, defined as Equation (5). A step-by-step numerical example illustrating the offspring angle calculation is provided in Appendix A for clarity.

(5) $$w_{p}=\frac{1}{f\left(X_{p}\right)+\varepsilon },{\;w}_{m}=\frac{1}{f\left(X_{m}\right)+\varepsilon }$$

To avoid numerical instability in cases where $f\left(X\right)$ approaches zero, a small positive constant ε can be added to the denominator when necessary.

Therefore, the more successful parent has a stronger influence on the offspring’s migration direction. The coefficients $\lambda_{1}$ and $\lambda_{2}$ control the angular deviation of each offspring from the mean route of the parents, and $\varepsilon_{1},\varepsilon_{2}$ are small random perturbations that reflect natural variability in orientation behavior. The goal of this mechanism is to generate controlled diversity in the initial directions of the new generation so that the two offspring move toward different regions of the search space.

After determining the migration direction, the movement of each bird in the search space is performed using its movement angle and step size. The position of individual i at iteration t+1 is updated by Equation (6).

(6) $$X_{i}^{t+1}=X_{i}^{t}+r_{i}^{t}\left[\begin{array}{c} cos(\theta_{i}^{t})\\ sin(\theta_{i}^{t}) \end{array}\right]+\kappa (X_{g}^{t}-X_{i}^{t})$$

In this relation, $X_{i}^{t}$ is the current position of individual i, $r_{i}^{t}$ is the step size, and $\theta_{i}^{t}$ is the migration angle at iteration t. The term $X_{g}^{t}$ is the global best position of the population at the same iteration, selected among all N individuals. The coefficient κ controls the strength of the general tendency of the population toward this position. This term guides the migration paths in the long run toward promising regions of the search space, while the main movement is still governed by the migration direction. As migration continues and iterations proceed, birds gradually adjust their routes. This direction correction is influenced by collective experience, inherited tendencies, and previously successful positions. The migration angle of individual i at iteration t+1 is updated by Equation (7).

(7) $$\theta_{i}^{t+1}=\theta_{i}^{t}+\beta_{1}(\theta_{g}^{t}-\theta_{i}^{t})+\beta_{2}(\theta_{n}^{t}-\theta_{i}^{t})+\sigma_{i}^{t}$$

where $\theta_{g}^{t}$ is the movement angle of the global best individual in the population, $\theta_{n}^{t}$ is the movement angle of a successful neighbor, and $\beta_{1}\;and\;\beta_{2}$ are coefficients that control collective direction adjustment. The term $\sigma_{i}^{t}$ is an adaptive disturbance, defined by Equation (8).

(8) $$\sigma_{i}^{t}=\xi_{i}^{t}\;exp(-t/T)$$

where $\xi_{i}^{t}$ is a random variable and T is a time-scale parameter. This structure makes the magnitude of angular fluctuations larger in the early stages of the search and gradually smaller as the number of iterations increases. The successful neighbor is selected from the local neighborhood of the current individual. Among the candidate neighbors, the individual with the best fitness value is regarded as the successful neighbor, and its movement angle is used in the angle update mechanism. Therefore, the selection is based on objective function improvement rather than spatial distance alone. If no neighboring candidate achieves a better fitness value than the current individual, the previously identified successful neighbor is retained to maintain directional stability in the migration process.

One of the most important biological features in migratory behavior is the resistance of resident birds to the entry of migrants into established territories. When a migratory bird enters a region of the search space already occupied by another bird, a kind of territorial competition occurs. If the migrant has worse fitness, it is rejected by the resident bird and forced to change its route. In the model, this mechanism is implemented as follows. First, the acceptance probability of the migrant in the resident’s territory is defined by Equation (9).

(9) $$P_{\mathrm{accept}}=\frac{f(X_{i}^{t})}{f(X_{i}^{t})+f(X_{s}^{t})}$$

where $X_{i}^{t}$ is the position of the migrant, $X_{s}^{t}$ is the position of the resident bird, and $f\left(\cdot \right)$ is the objective function. If a random number $u∼U\left(0,1\right)$ is greater than $P_{\mathrm{accept}}$, the migrant is rejected, and its movement angle is modified by Equation (10).

(10) $$\theta_{i}^{t+1}=\theta_{i}^{t+1}+\delta_{1}R_{1}+\delta_{2}\frac{X_{i}^{t}-X_{s}^{t}}{∥X_{i}^{t}-X_{s}^{t}∥}$$

where $R_{1}$ is a random variable, and $\delta_{1}\;and\;\delta_{2}$ are coefficients that control the deviation intensity. The second term models the repulsion of the migrant from the occupied territory and forces its path to deviate toward neighboring regions.

Finally, after applying all stages of movement, angle correction, territorial interactions, and reproduction in each iteration, the objective function is evaluated for all individuals, and the best position of the population is updated by Equation (11).

(11) $$X_{g}^{t+1}=arg\;\underset{i}{min}f(X_{i}^{t+1})$$

Here, $X_{g}^{t+1}$ is the best solution obtained at iteration t+1 among all individuals. The corresponding angle of this position is used as a reference direction for the population in the next iterations. In this way, the search process gradually moves toward the optimal regions of the problem space.

**Figure 3:**
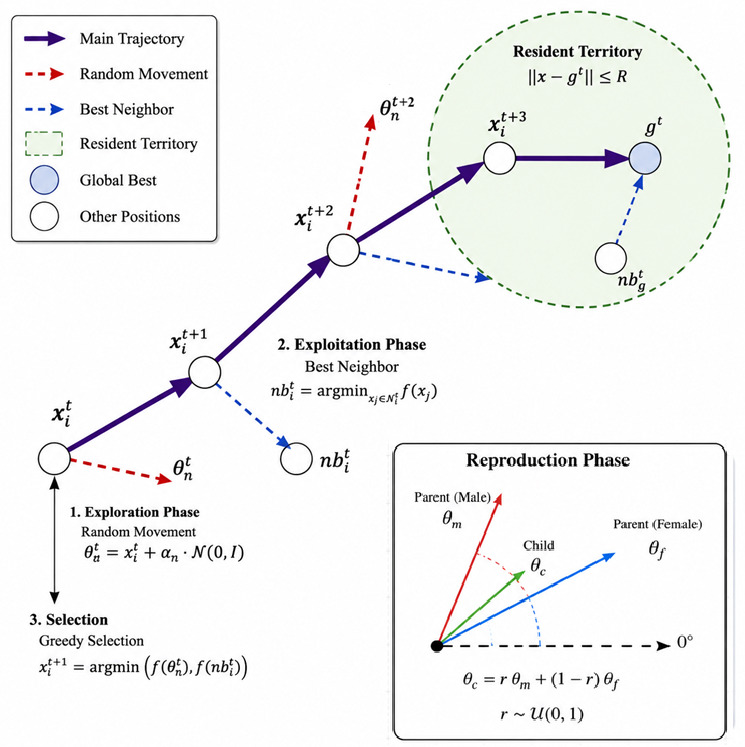
Movement mechanism in the BCOA.

Figure 3 provides a comprehensive illustration of the angle-based movement mechanism in the BCOA. In this model, the main movement output of each bird in every iteration is determined by the position update equation in Equation (12).

(12) $$x_{i}^{\left(t+1\right)}=x_{i}^{\left(t\right)}+s_{i}^{\left(t\right)}\;d(\theta_{i}^{\left(t\right)})+\alpha \;(g^{\left(t\right)}-x_{i}^{\left(t\right)})$$

where the bird moves forward using the step length $s_{i}^{\left(t\right)}$ and the direction vector associated with its movement angle $d(\theta_{i}^{\left(t\right)})$, while being simultaneously attracted toward the global best position $g^{\left(t\right)}$ through the coefficient α. The future movement angle of the bird, $\theta_{i}^{\left(t+1\right)}$, is updated using Equation (13).

(13) $$\theta_{i}^{\left(t+1\right)}=\theta_{i}^{\left(t\right)}+\beta_{1}(\theta_{g}^{\left(t\right)}-\theta_{i}^{\left(t\right)})+\beta_{2}(\theta_{n}^{\left(t\right)}-\theta_{i}^{\left(t\right)})+\varepsilon_{i}^{\left(t\right)}$$

In this geometric process, the new angle is corrected under the influence of the global best angle $\theta_{g}^{\left(t\right)}$ and the angle of a successful neighbor $\theta_{n}^{\left(t\right)}$. The term $\varepsilon_{i}^{\left(t\right)}=\gamma \;\xi_{i}\;(1-t/T)$ acts as an adaptive random disturbance that maintains the exploration capability of the algorithm. Beyond the main movement path, the diagram also shows the effect of the territorial-competition mechanism. When a bird enters the territory of a resident bird, a final correction (Resident Influence) is applied to its movement direction, which stabilizes the final position $x_{i}^{\left(t+2\right)}$. Finally, the reproduction phase in the figure represents the angle-inheritance relation by Equation (14).

(14) $$\theta_{\mathrm{child}}=w_{m}\theta_{m}+w_{f}\theta_{f}+\delta$$

in which the migration angles of the male and female parents are combined using fitness-based weights $\left(w_{m},w_{f}\right)$, and small random deviations δ are added to preserve angular diversity in the next generations.

To provide a clearer understanding of the state-transition mechanism in the BCOA, the response matrix (the new population positions at iteration t+1) can be expressed as the sum of the previous state matrix and the change matrix (movement steps and global-best tendency). Let N be the number of birds and D the dimension of the problem space:

$$\left[\begin{array}{cccc} x_{1,1}^{t+1} & x_{1,2}^{t+1} & \cdots & x_{1,D}^{t+1}\\ x_{2,1}^{t+1} & x_{2,2}^{t+1} & \cdots & x_{2,D}^{t+1}\\ \vdots & \vdots & \ddots & \vdots \\ x_{N,1}^{t+1} & x_{N,2}^{t+1} & \cdots & x_{N,D}^{t+1} \end{array}\right]=\left[\begin{array}{cccc} x_{1,1}^{t} & x_{1,2}^{t} & \cdots & x_{1,D}^{t}\\ x_{2,1}^{t} & x_{2,2}^{t} & \cdots & x_{2,D}^{t}\\ \vdots & \vdots & \ddots & \vdots \\ x_{N,1}^{t} & x_{N,2}^{t} & \cdots & x_{N,D}^{t} \end{array}\right]+\left[\begin{array}{cccc} \Delta x_{1,1}^{t} & \Delta x_{1,2}^{t} & \cdots & \Delta x_{1,D}^{t}\\ \Delta x_{2,1}^{t} & \Delta x_{2,2}^{t} & \cdots & \Delta x_{2,D}^{t}\\ \vdots & \vdots & \ddots & \vdots \\ \Delta x_{N,1}^{t} & \Delta x_{N,2}^{t} & \cdots & \Delta x_{N,D}^{t} \end{array}\right]$$

In this formulation, the change matrix $\Delta X^{\left(t\right)}$ is derived from the angular and positional components of the previous state. Each entry $\Delta x_{i,j}^{\left(t\right)}$, representing the position change in bird i in dimension j, is obtained directly from the angle-based relations, shown by Equation (15).

(15) $$\Delta x_{i,j}^{\left(t\right)}=s_{i}^{\left(t\right)}\;d_{j}(\theta_{i}^{\left(t\right)})+\alpha \;(g_{j}^{\left(t\right)}-x_{i,j}^{\left(t\right)})$$

Similarly, the response matrix for the movement angles of the flock, $\Theta^{\left(t+1\right)}$, which determines the future orientations of individuals, is produced through an element-wise update of the previous angle matrix:

$$\left[\begin{array}{ccc} \theta_{1,1}^{t+1} & \cdots & \theta_{1,D}^{t+1}\\ \vdots & \ddots & \vdots \\ \theta_{N,1}^{t+1} & \cdots & \theta_{N,D}^{t+1} \end{array}\right]=\left[\begin{array}{ccc} \theta_{1,1}^{t} & \cdots & \theta_{1,D}^{t}\\ \vdots & \ddots & \vdots \\ \theta_{N,1}^{t} & \cdots & \theta_{N,D}^{t} \end{array}\right]+\left[\begin{array}{ccc} \Delta \theta_{1,1}^{t} & \cdots & \Delta \theta_{1,D}^{t}\\ \vdots & \ddots & \vdots \\ \Delta \theta_{N,1}^{t} & \cdots & \Delta \theta_{N,D}^{t} \end{array}\right]$$

Each angular-deviation entry $\Delta \theta_{i,j}^{\left(t\right)}$ is produced by the simultaneous influence of the global-best angle, the angle of a successful neighbor, and the random disturbance term $\varepsilon_{i,j}^{\left(t\right)\;}$ in the same dimension by Equation (16).

(16) $$\Delta \theta_{i,j}^{\left(t\right)}=\beta_{1}\;(\theta_{g,j}^{\left(t\right)}-\theta_{i,j}^{\left(t\right)})+\beta_{2}\;(\theta_{n,j}^{\left(t\right)}-\theta_{i,j}^{\left(t\right)})+\varepsilon_{i,j}^{\left(t\right)}$$

Algorithm 1 shows the process of the BCOA.
**Algorithm 1:** Blackcap Optimization Algorithm (BCOA)Inputs: N, $Max_{Iter}$, s, d, Boundaries of the search space, α, $\beta_{1}$, $\beta_{2}$, γ, and step size s
Outputs: g: Global best position, $F_{best}$: best solution Fitness value
1. For each bird i from 1 to N do:2. Initialize random position $x_{i}^{0}$ within the search space boundaries3. Initialize random migration angle $\theta_{i}^{0}$4. Evaluate fitness value $f(x_{i}^{0})$5. End For6. Find the best bird and store its position as $g^{0}$ and its angle as $\theta_{g}^{0}$7. Set iteration counter t=08. While (t<Max_Iter) do:9. For each bird i from 1 to N do:10. Select two parents with angles $\theta_{m}$ and $\theta_{f}$, and fitness weights $w_{m}$ and $w_{f}$11. Calculate the weighted average of parents’ angles:            ${\bar{\theta }}_{parents}=w_{m}\theta_{m}+w_{f}\theta_{f}$12. Calculate migration angles for offspring by applying random perturbations ($\delta_{1},\delta_{2}$):              $\theta_{child_{1}}={\bar{\theta }}_{parents}+\delta_{1}$              $\theta_{child_{2}}={\bar{\theta }}_{parents}+\delta_{2}$13. Select the angle of a successful neighbor $\theta_{n}^{t}$14. Calculate the decaying adaptive perturbation                 $\xi_{i}^{t}=r_{i}^{t}\cdot e^{-\gamma t}$15. Select the angle of a successful neighbor $\theta_{n}^{t}$16. $n^{*}=arg{min}_{j\in N(i)}f(x_{j}^{t}),\theta_{n}^{t}=\theta_{n^{*}}^{t}$17. Update the bird’s angle based on the global best, successful neighbor, and perturbation:          $\theta_{i}^{t+1}=\theta_{i}^{t}+\beta_{1}(\theta_{g}^{t}-\theta_{i}^{t})+\beta_{2}(\theta_{n}^{t}-\theta_{i}^{t})+\xi_{i}^{t}$18. Determine the direction vector $d\left(\theta_{i}^{t}\right)$ based on the current migration angle19. Update the position in the search space:          $x_{i}^{t+1}=x_{i}^{t}+s_{i}^{t}\cdot d(\theta_{i}^{t})+\alpha (g^{t}-x_{i}^{t})$20. If the new position $x_{i}^{t+1}$ goes beyond $\left[Lower\_Bound,Upper\_Bound\right]$, amend it.21. End For22. For each bird i from 1 to N do:23. Evaluate fitness value $f(x_{i}^{t+1})$24. If $f\left(x_{i}^{t+1}\right)$ is better than the global best fitness ($F_{best}$) then:25. Update the global best position: $g^{t+1}=x_{i}^{t+1}$26. Update the best bird’s angle: $\theta_{g}^{t+1}=\theta_{i}^{t+1}$27. Update $F_{best}=f(x_{i}^{t+1})$28. End If29. End For30. t=t+131. End While32. Return g and $F_{best}$


To provide a more precise understanding of the execution procedure of the BCOA, the pseudocode structure can be explained as a logical and continuous process. The algorithm begins in the initial lines by setting the parameters and randomly generating an initial population of birds. For each individual i, an initial position vector $x_{i}$ and an initial migration angle $\theta_{i}$ are defined in the search space of the problem. After evaluating the fitness of all individuals, the position and angle of the bird with the best fitness are identified as the global leader $\left(g,\theta_{g}\right)$ and stored in memory.

Upon entering the main loop of the algorithm, the evolutionary process in each iteration starts with the generation and inheritance mechanism. In this stage, birds are selected as parents (male and female) according to their fitness values, and the migration angle of each new offspring is inherited by calculating the weighted average of the parental angles, $\left(w_{m}\theta_{m}+w_{f}\theta_{f}\right)$, combined with a small random disturbance $\left(\delta \right)$. This structure ensures that the information of successful routes is transferred from one generation to the next, while the genetic diversity of the population is preserved at the same time.

In the next stage, which acts as the core of the algorithm, the adaptive angle-updating mechanism is executed. In these lines, each bird adjusts its next movement angle $\theta_{i}^{\left(t+1\right)}$ under the influence of three key factors: the tendency to move toward the angle of the current global best individual $\left(\theta_{g}\right)$, the influence of the angle of a successful neighbor $\left(\theta_{n}\right)$, and the application of a decreasing search fluctuation $\left(r_{i}^{\left(t\right)}e^{-\gamma t}\right)$. The inclusion of the exponential term $e^{-\gamma t}$ in this equation is very important because as the iteration number t increases, its value gradually decreases. As a result, the algorithm shows an intelligent behavior: in the early iterations, it focuses more on the global exploration phase, while in the final iterations, it smoothly shifts toward the local exploitation phase.

Finally, after the new movement angle is determined, the position-updating step is performed in the problem space. In the final lines of the loop, the position of each bird, $x_{i}^{\left(t+1\right)}$, is updated based on the new direction vector $d(\theta_{i}^{\left(t\right)})$, the step size $s_{i}^{\left(t\right)}$, and a controlled attraction force toward the global best position g. After these movements are completed, the fitness of the new positions is evaluated again, and the global best individual g is updated for use in the next iteration. This cycle of evaluation and continuous correction continues until the stopping condition, namely the maximum number of iterations, is satisfied. At the end, the best position discovered during the entire search process is returned as the final solution of the problem. Table 1 presents the symbols used in the study along with their descriptions.

## 4. Evaluation

In this section, the proposed BCOA is evaluated under unimodal and multimodal benchmarks and real-world optimization problems. Also, some performance metrics are deployed to show the BCOA efficiency.

### 4.1. Experiment Setup and Compared Algorithms

The experiments were conducted on a system running Windows 11 equipped with an Intel Core i7 processor at 2.8 GHz and 16 GB of RAM DDR4. The processor was manufactured by Intel Corporation (USA). All implementations of the algorithms were carried out in Python 3.14.6, using the Anaconda distribution and the Jupyter Notebook 7.5 environment. Several common Python libraries were used in the development process, including NumPy for numerical computations, SciPy for mathematical functions and optimization tasks, Matplotlib 3.10.6 for plotting, and the random module for generating stochastic values. All experiments were executed under the same computational conditions to ensure a consistent evaluation platform and to maintain fair comparability among the algorithms.

To ensure a fair and comprehensive evaluation, nine metaheuristic algorithms from four major categories were selected, incorporating recent state-of-the-art methods. The selection includes two foundational classical algorithms—Genetic Algorithm (GA) [14] and Particle Swarm Optimization (PSO) [35]—which serve as established benchmark references. To reflect the progress of modern metaheuristic research, seven high-performance algorithms were included: Heterogeneous Alternating Evolutionary Algorithm (HAEA) [15], the Adaptive Competitive Swarm Optimizer (AMRCSO) [36], Whale Optimization Algorithm (WOA) [41], Kepler Optimization Algorithm (KOA) [69], Chef-Based Optimization Algorithm (CBOA) [96], the L-AL-SHADE algorithm [101], and the Polar Fox Optimization (PFO) algorithm [102]. These methods represent a diverse range of computational inspirations, including evolution, swarm intelligence, physics, and migration-based strategies, ensuring a robust assessment of BCOA’s performance.

To evaluate the performance of the algorithms, several common statistical indicators were employed, including the best-obtained value (Best), the worst value (Worst), the mean value across all runs (Mean), and the standard deviation (Std). These metrics provide a comprehensive assessment of solution quality, algorithmic stability, and result consistency. Furthermore, to ensure fairness in comparison, the control parameters of each algorithm were configured according to the recommended settings reported in their respective reference papers. For the proposed BCOA, the control parameters were assigned based on the values introduced in Section 3. All experiments were conducted under identical computational conditions to guarantee a uniform testing environment for all algorithms.

To further evaluate the reliability of the algorithms, the success rate (SR) is also reported. The success rate represents the percentage of runs that reach a solution within a tolerance of 10^−6^ from the best-obtained value. It is defined as SR = (Ns/30) × 100, where Ns denotes the number of successful runs. This metric provides an additional measure of convergence reliability.

### 4.2. Benchmark Functions

To provide a rigorous evaluation of the proposed algorithm, a set of standard benchmark functions widely used in optimization research was employed. These functions are commonly used for assessing metaheuristic algorithms because of their diverse characteristics, including numerous local optima, complex search landscapes, and varying problem dimensionalities. The use of such benchmark functions enables a fair and reliable comparison between the proposed method and existing optimization algorithms.

In this study, the benchmark functions are divided into two main categories: unimodal and multimodal functions. Unimodal functions generally contain a single global optimum and are mainly used to evaluate the convergence behavior and exploitation capability of optimization algorithms. In contrast, multimodal functions include multiple local optima and are therefore suitable for evaluating the exploration ability of algorithms within the search space.

Each benchmark function is characterized by a specific range for its decision variables, a predefined problem dimension, and a known global optimum value. To ensure a fair comparison, all algorithms were executed under identical experimental conditions. Detailed information about the benchmark functions, including the function name, variable range, problem dimension, and global optimum value, is presented in Table 2 and Table 3. These benchmark functions have been extensively used in previous studies for evaluating metaheuristic optimization algorithms. Therefore, the obtained results provide a reliable basis for assessing the effectiveness and competitiveness of the proposed Blackcap Optimization Algorithm (BCOA) compared with other optimization methods.

### 4.3. Results on Benchmark Functions

In this section, the performance of the proposed Blackcap Optimization Algorithm (BCOA) is evaluated on the set of benchmark functions introduced in the previous section. The main objective of these experiments is to investigate the ability of the algorithm to obtain optimal solutions, its stability, and the quality of its convergence behavior compared with other well-known metaheuristic algorithms. To obtain reliable statistical results, each algorithm was independently executed 30 times on each benchmark function. Then, three statistical indicators, including Best, Mean, and Standard Deviation (Std), were reported. The Best value represents the best objective function value obtained among all runs, Mean indicates the average performance of the algorithm, and Std reflects the stability and variation in the results. The proposed BCOA was compared with nine well-known algorithms, including GA, PSO, AL-SHADE, HAEA, AMRCSO, WOA, KOA, CBOA and PFO. The obtained results are categorized into two groups. The first group includes unimodal functions (F1–F16), which are used to evaluate the exploitation ability of the algorithms. The second group includes multimodal functions (F17–F32), which are used to assess their exploration ability. Table 4 presents the optimal parameter settings of the algorithms used in the experiments.

Parameter tuning plays an important role in the performance of metaheuristic algorithms. Therefore, a sensitivity analysis was conducted to investigate the effect of the main control parameters of BCOA, including α, β, γ, and the step size s. Each parameter was varied within a reasonable range while keeping the other parameters fixed. Table 5 summarizes the tested values and the selected settings. The experimental observations show that moderate values of these parameters provide a better balance between exploration and exploitation. In particular, the combination α = 0.4, β = 0.6, γ = 0.1, and s = 0.5 produced the most stable and competitive performance across the benchmark functions. These values were therefore adopted in all subsequent experiments.

The results presented in Table 6 and Table 7 show the performance of the BCOA on the unimodal functions (F1–F16) and multimodal functions (F17–F32). As observed, the algorithm achieves better Mean and Best values than the comparative methods on a considerable number of functions, and in many cases, it also provides lower Std values. This behavior can be directly attributed to the angle-based search strategy and the internal control mechanisms embedded in the structure of BCOA. In BCOA, the movement direction of each individual is determined by the migration angle, and the new position of each bird is updated based on this angle and the corresponding movement step. This directional movement model ensures that the search is guided rather than being purely random in the coordinate space.

For unimodal functions, this property, along with the attraction toward the global best position X_best, enables the search trajectories to gradually concentrate around the optimal region. As a result, the population moves coherently toward a dominant direction in the search space, leading to fast and stable convergence, which is reflected in the low Mean and Std values reported in the unimodal function results. Furthermore, during the reproduction phase, the migration angle of each offspring is generated through a weighted combination of the parents’ angles. The weights are assigned based on the fitness values, meaning that birds with better performance have a stronger influence on determining the movement direction of the next generation. This operator reinforces successful search directions and helps preserve efficient exploration paths across generations. In unimodal problems, this mechanism accelerates the convergence toward the optimal region and reduces the overall variability of the obtained solutions.

For multimodal functions, where the presence of many local optima may lead to premature convergence, two additional mechanisms of BCOA play an essential role. The first is the adaptive perturbation applied to the migration angle, denoted by γ(t). At the early stages of the search, γ(t) takes relatively large values, generating broader directional changes in the birds’ movement paths. These large directional variations help the population escape from local regions and explore different parts of the search space. As the number of iterations increases, γ(t) gradually decreases, making the movements more stable and focused. This process supports a smooth transition from exploration to exploitation. The second mechanism is the territorial competition strategy, which prevents excessive accumulation of individuals in the same region of the search space. In this mechanism, if a migrating bird performs worse than the resident bird occupying a territory, its movement direction is modified, and it is pushed away from that area. This operator forces weaker individuals to move toward new regions, promoting a diverse population distribution. Consequently, the likelihood of falling into local optima decreases, and the global search capability is enhanced, as reflected in the competitive performance of BCOA on the multimodal functions reported in Table 6.

Overall, the combination of four main components—the angle-based directional movement, the guided transfer of successful directions through parent-angle recombination, the adaptive control of angular variations, and the territorial competition mechanism—enables BCOA to maintain an effective balance between exploration and exploitation. This balanced behavior results in competitive and stable performance across both unimodal and multimodal benchmark functions.

#### 4.3.1. Average Convergence Analysis

To examine the dynamic behavior of the proposed algorithm, convergence graphs were used as one of the common tools for evaluating optimization performance in metaheuristic studies. A convergence graph shows the variation in the best objective value obtained by an algorithm during the iterative search process. In other words, these graphs visualize how algorithms approach the optimal solution throughout the optimization process. Using this metric allows a more detailed analysis of convergence speed, exploitation ability, and stability of algorithms. While statistical results such as the mean and standard deviation only describe the final performance of an algorithm, convergence curves reveal the trajectory of the search process, allowing researchers to observe and compare algorithm behavior in the early, mid, and final stages of optimization. Therefore, convergence analysis provides a deeper understanding of the balance between global search (exploration) and local search (exploitation) capabilities in different algorithms.

**Figure 4:**
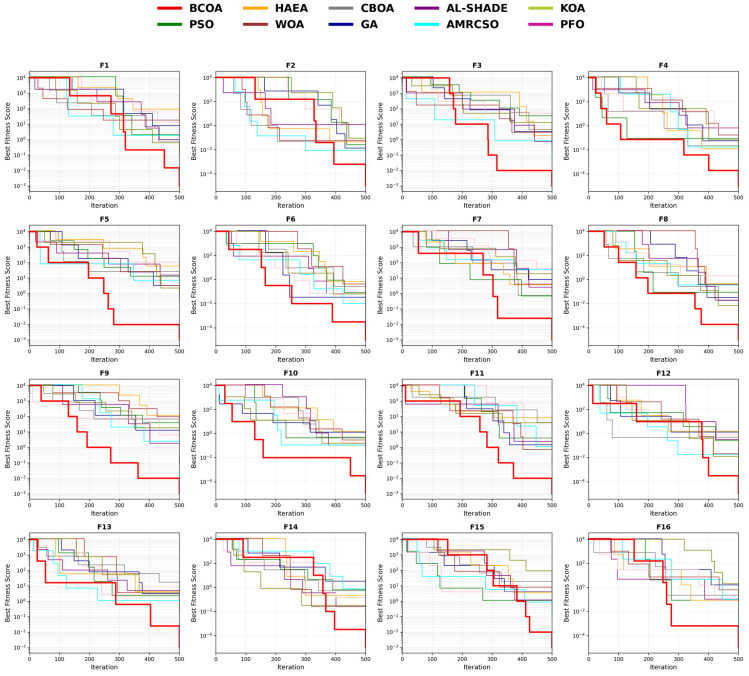
Average Convergence Analysis of unimodal benchmark functions.

To evaluate the performance of the proposed BCOA more precisely, its convergence curves were compared against other well-known metaheuristic methods on two groups of standard functions. Figure 4 shows the convergence behavior on unimodal functions (F_1_–F_16_). Since unimodal functions contain only one global optimum and no local optima, they serve as an appropriate benchmark to test exploitation capability and local search accuracy. As seen in Figure 4, the convergence curve of BCOA (bold line) descends much more steeply than the other competitors and quickly approaches near-zero values in the early iterations. This behavior demonstrates the high convergence speed and precise position-updating mechanisms of BCOA, enabling it to move quickly and accurately toward the global optimum once the promising region is located. In contrast, other algorithms converge more slowly and stop at higher error levels.

**Figure 5:**
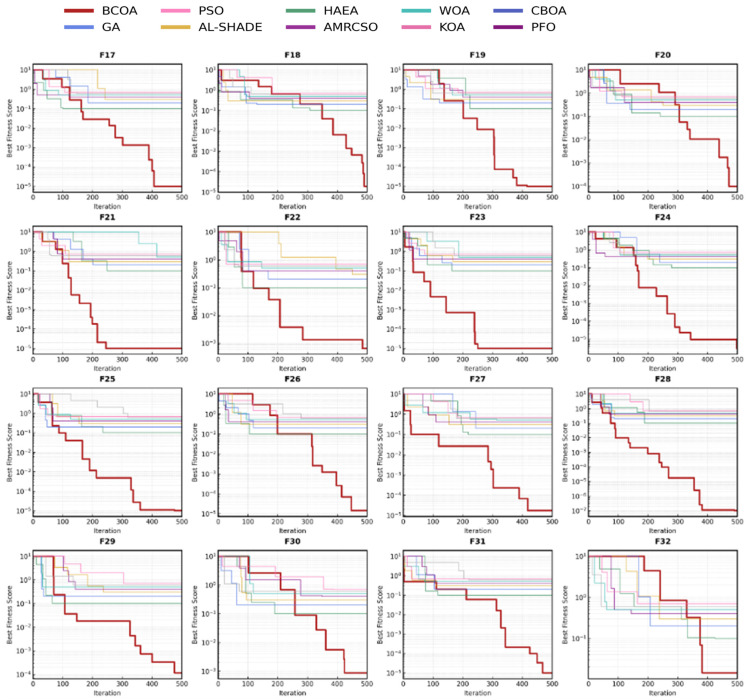
Average Convergence Analysis of multimodal benchmark functions.

However, the main challenge of optimization algorithms when dealing with complex real-world problems is managing rough search spaces—represented here by the multimodal functions (F_17_–F_32_) shown in Figure 5. Multimodal functions include deceptive valleys and numerous local optima, which severely test the algorithm’s exploration capability and its ability to escape local traps. The analysis of Figure 5 clearly confirms the superior performance of BCOA compared with other methods. As illustrated in these graphs, most competing algorithms (such as PSO, GWO, and other classical methods) lose population diversity too early and experience premature convergence. Their curves flatten at high objective values after a slight initial drop, indicating that they remain trapped in local optima and fail to improve until the end of the run.

The main reason behind BCOA’s strong performance on multimodal functions (Figure 5) lies in its dynamic and intelligent balance between exploration and exploitation phases. The rapid early decline of the BCOA curve toward lower objective values (around iterations 100–150) indicates that the algorithm does not waste time exploring unpromising regions. The unique mechanisms of BCOA—including the reproduction phase and the adaptive angle-updating process described earlier in the pseudocode—continuously inject momentum into the population and prevent stagnation in local traps. This structure enables BCOA not only to identify and escape from local optima quickly but also to intensify exploitation once it enters the basin of the global optimum. As a result, while other methods continue struggling with poor local solutions even in the final iterations (around iteration 400 or later), BCOA reaches the lowest error level within the first third of the run and maintains stable convergence until the end. This characteristic makes BCOA a fast, robust, and reliable optimizer for highly complex and multimodal search spaces.

Since metaheuristic algorithms are inherently stochastic, the result of a single run cannot fully represent their actual performance. Therefore, to provide a more accurate and reliable evaluation, average convergence curves based on multiple independent runs are examined. In this study, each algorithm was executed several times independently, and the mean objective value at each iteration was calculated across all runs. The average convergence curve represents the overall progress of an algorithm during the optimization process and reduces the influence of randomness that exists in population-based methods. This metric is widely used in scientific studies because it offers a clearer view of the general convergence trend, the stability of the algorithm, and the reliability of its performance. By considering the mean performance over multiple runs, the real behavior of an algorithm can be assessed more accurately.

#### 4.3.2. Execution Time Analysis

In addition to solution quality and convergence behavior, the computational efficiency of optimization algorithms is also an important criterion for evaluating their performance. In many real-world optimization problems, especially large-scale or time-sensitive applications, algorithms must not only provide high-quality solutions but also do so within a reasonable computation time. Therefore, analyzing the execution time of algorithms offers useful insight into their computational complexity, efficiency, and scalability. An algorithm that can reach competitive or better results in less time is generally considered more suitable for practical applications.

To evaluate the computational efficiency of the proposed method, the execution time of BCOA was compared with several well-known metaheuristic algorithms across all benchmark functions. All experiments were performed under identical conditions to ensure a fair comparison. The results show that the proposed BCOA has notable computational efficiency and, in most cases, achieves shorter execution times than the other algorithms. This improvement can be attributed to its effective search strategy and the balanced interaction between exploration and exploitation, which help the algorithm move quickly toward promising regions of the search space and avoid unnecessary computations. More specifically, BCOA demonstrates faster execution times on most unimodal and multimodal functions compared with many of the competing methods. The reduced computation time indicates that the algorithm requires fewer operations to guide the search process. The relatively stable execution time across different functions also shows that the algorithm maintains consistent computational behavior when dealing with varying levels of problem complexity.

Overall, the results indicate that the proposed BCOA not only provides strong optimization performance but also performs well in terms of computational efficiency. This combination of high-quality solutions and shorter execution times makes BCOA a suitable choice for a wide range of optimization problems, especially in applications where time constraints are critical. Table 8 and Table 9 present the execution times of the algorithms on the benchmark functions.

#### 4.3.3. Statistical Test Analysis

To statistically evaluate the performance of the proposed algorithm, the non-parametric Wilcoxon signed-rank test and Friedman and Nemenyi Statistical Analysis were employed [103].

##### Wilcoxon Signed-Rank Test

This test is one of the most commonly used methods for comparing metaheuristic optimization algorithms because, unlike parametric tests, it does not require the assumption of normal data distribution. Considering the stochastic nature of metaheuristic algorithms and the variability of their results across different runs, the Wilcoxon test provides a reliable and accurate way to compare the performance of different methods. In this study, the test was conducted at a significance level of 0.05 to examine the statistical significance of the performance differences between the proposed BCOA and the other compared algorithms. In the reported tables, the symbol “+” indicates that BCOA performs significantly better than the compared algorithm, the symbol “=” indicates no statistically significant difference, and the symbol “−” indicates that the proposed algorithm performs worse than the corresponding competitor.

Table 10 presents the Wilcoxon test results for the unimodal functions (F1–F16). These functions are mainly used to evaluate the exploitation ability and convergence speed of optimization algorithms. The results of this table show that the proposed BCOA performs better than the reference algorithms in most comparisons and, in many cases, achieves statistically significant superiority over them. The large number of “+” signs indicates that BCOA is able to converge toward the optimal solution with high accuracy and possesses strong exploitation capability. At the same time, the presence of a limited number of “=” results indicates that, for some functions, the performance of the proposed algorithm is statistically similar to that of some competing methods. Overall, the results of this table demonstrate the strong ability of BCOA to solve unimodal problems and to reach high-quality solutions efficiently.

Table 11 reports the Wilcoxon test results for the multimodal functions (F17–F32). These functions are more challenging because of the presence of multiple local optima and are mainly used to assess the exploration ability and global search performance of algorithms. According to the results, BCOA still shows competitive and remarkable performance compared with the other algorithms. In many cases, BCOA significantly outperforms the compared algorithms, as indicated by the “+” sign. For some functions, the performance differences are not statistically significant and are therefore marked with “=”. In a very limited number of cases, a competing algorithm may show better performance, which is indicated by the “−” sign. Nevertheless, the overall trend of the results shows that the proposed algorithm maintains a proper balance between exploration and exploitation and is capable of finding suitable solutions even in complex search spaces.

Table 12 provides a summary of the Win/Tie/Loss results obtained from the statistical comparison between BCOA and the competing algorithms. This table reports the number of cases in which the proposed algorithm performs better (Win), similarly (Tie), or worse (Loss) than each competing method. Since all comparisons are made with respect to BCOA, the name of this algorithm is not listed separately in the table. The results show that BCOA achieves a higher number of wins against most of the compared algorithms, while the number of ties is limited and the number of losses is very small. These findings indicate that the proposed algorithm has high stability and efficiency and, overall, provides competitive and often superior performance compared with existing methods. Therefore, the results of the Wilcoxon statistical test further confirm the effectiveness and reliability of the proposed algorithm for solving different optimization problems.

##### Friedman and Nemenyi Statistical Analysis

To provide a rigorous statistical validation of the experimental results, the Friedman non-parametric test is employed to compare the proposed BCOA with the competing algorithms over all 32 benchmark functions. The Friedman test is particularly suitable in this context because it does not assume normality of the performance samples and is designed to compare multiple algorithms across multiple test problems. For each benchmark function, the algorithms are ranked according to their mean objective values, where rank 1 is assigned to the best-performing algorithm. Let k denote the number of algorithms and N denote the number of benchmark functions. The Friedman statistic is computed as follows.

$$\chi_{F}^{2}=\frac{12N}{k(k+1)}\sum_{j=1}^{k}R_{j}^{2}-3N(k+1)$$

where $R_{j}$ is the average rank of the j-th algorithm across all benchmark functions. If the null hypothesis is rejected, a Nemenyi post hoc test is performed to determine whether the rank differences between two algorithms are statistically significant. The critical difference is defined as

$$CD=q_{\alpha }\sqrt{\frac{k(k+1)}{6N}}$$

where $q_{\alpha }$ is the critical value from the Studentized range distribution at the selected significance level. When the absolute difference between the average ranks of two algorithms exceeds the critical difference, their performance difference is considered statistically significant. Table 13 presents the ranking results obtained from the Friedman statistical test for all algorithms on the 32 benchmark functions (F1–F32). In this table, the algorithms are ranked for each benchmark function according to their mean performance values, where rank 1 represents the best-performing algorithm and higher ranks indicate lower performance. This ranking process allows a fair comparison of the competing algorithms across all benchmark problems.

Table 14 summarizes the average ranks of the algorithms computed from the Friedman test results across the entire set of benchmark functions. The average rank is calculated by taking the mean of the individual ranks obtained by each algorithm over all functions. This table provides an overall view of the relative performance of the algorithms and is commonly used to determine the global ranking before applying post hoc statistical comparisons such as the Nemenyi test.

Overall, the results presented in Table 13 and Table 14 provide a comprehensive statistical comparison of the studied algorithms across the full benchmark suite. The ranking distribution across the benchmark functions shows consistent differences in performance among the algorithms. The average ranking results further highlight the overall effectiveness and stability of the methods when evaluated over both unimodal and multimodal optimization problems, indicating that the proposed approach demonstrates competitive and reliable performance across the tested scenarios.

##### Post Hoc Nemenyi Test

Since the Friedman test indicates differences in the performance of the compared algorithms, a post hoc analysis using the Nemenyi test is conducted to determine whether these differences are statistically significant. The Nemenyi test compares the average ranks of all algorithms pairwise. Two algorithms are considered significantly different if the difference between their average ranks exceeds the Critical Difference (CD) value.

The Critical Difference is calculated as

$$CD=q_{\alpha }\sqrt{\frac{k(k+1)}{6N}}$$

where k is the number of algorithms, N is the number of benchmark functions, and $q_{\alpha }$ is the critical value based on the Studentized range statistic at significance level α.

In this study, k=10 algorithms and N=32 benchmark functions were used. Using the standard significance level α=0.05, the computed Critical Difference (CD) is approximately 2.65.

The pairwise comparison based on the Nemenyi test shows that the differences between several algorithms exceed the critical difference, indicating statistically significant performance differences. These results confirm that the proposed algorithm demonstrates statistically competitive performance when evaluated over the entire benchmark suite.

In addition to significance testing, the Vargha–Delaney A measure is employed to quantify the magnitude of the performance difference between BCOA and the compared algorithms. The A value represents the probability that the proposed algorithm yields better results than a competitor. The interpretation of the Vargha–Delaney A statistic follows the common convention used in non-parametric effect size analysis. An A value of 0.5 indicates no difference between the two algorithms, while values greater than 0.5 show that BCOA is more likely to outperform the corresponding competitor. In general, values in the range of approximately 0.56 to 0.63 can be regarded as a small effect, values between 0.64 and 0.70 indicate a medium effect, and values above 0.71 suggest a large effect size.

As shown in Table 15, BCOA demonstrates medium to large effect sizes against most of the compared algorithms, indicating that the observed superiority is not only statistically significant but also practically meaningful. In particular, the large A values obtained against several baseline methods confirm the strong competitive behavior of BCOA across the benchmark suite.

##### Ablation Analysis of BCOA Components

To assess the contribution of individual mechanisms, an ablation study was conducted by removing each core component of BCOA separately while keeping all other settings unchanged. The evaluated variants include: without angle-based movement, without adaptive disturbance, without territorial competition, and without reproduction/inheritance. Following the Friedman ranking procedure, the average ranks over the 32 benchmark functions were computed as

$$R_{j}=\frac{1}{32}\sum_{i=1}^{32}r_{ij}$$

where $r_{ij}$ denotes the rank of algorithm j on function i.

Table 16 presents the ablation analysis results using the average rank metric. The full BCOA achieves the best overall rank, indicating that the integration of all mechanisms leads to superior performance. Removing the angle-based movement causes the largest performance degradation, highlighting its key role in guiding the search process. The absence of adaptive disturbance and territorial competition also reduces performance, demonstrating their importance in maintaining the exploration–exploitation balance of the algorithm.

### 4.4. Real-World Engineering Problems

In this section, seven engineering optimization problems are considered to evaluate the performance of the proposed algorithm in practical applications. The first four problems are classical and widely used benchmark cases in the engineering optimization literature, commonly employed for evaluating and comparing metaheuristic algorithms. The remaining three problems are selected from modern and application-oriented domains related to computational systems and smart networks.

Since the engineering design problems considered in this section are constrained optimization problems, a constraint handling mechanism is incorporated into the BCOA framework to properly manage infeasible solutions during the optimization process. In this study, a penalty-based constraint handling strategy is adopted. Instead of discarding infeasible solutions, constraint violations are incorporated into the objective function through a quadratic penalty term. The penalized fitness function is defined as

$$F(x)=f(x)+\lambda \sum_{i=1}^{m}max(0,g_{i}(x){)}^{2}$$

where $f\left(x\right)$ denotes the original objective function, $g_{i}\left(x\right)\leq 0$ represents the i-th inequality constraint, m is the total number of constraints, and λ>0 is a penalty coefficient controlling the severity of constraint violations. To explicitly evaluate feasibility, the total constraint violation of a candidate solution is computed as

$$CV(x)=\sum_{i=1}^{m}max(0,g_{i}(x))$$

A solution is considered feasible when CV(x)=0; otherwise, it is regarded as infeasible. During the optimization process, BCOA minimizes the penalized fitness function F(x), which simultaneously drives the search toward feasible and optimal regions of the search space. All final solutions reported for the engineering case studies were verified using the defined constraint violation metric, confirming that the obtained optimal solutions satisfy all problem constraints.

Table 17 shows the problem parameters and their optimal values. To determine appropriate parameter settings for the proposed algorithm, the Taguchi method [104] was employed due to its efficiency in analyzing the effects of multiple parameters with a limited number of experiments. In this approach, several key control parameters of the algorithm were selected, and different levels were defined for each parameter. An appropriate orthogonal array was then used to systematically design the experimental runs, allowing the influence of each parameter on the algorithm’s performance to be evaluated. The performance of each configuration was assessed based on the objective function values obtained from the benchmark problems. Subsequently, the signal-to-noise (S/N) ratio was calculated to identify the parameter levels that provide robust and stable performance. Based on the analysis results, the optimal combination of parameter settings was determined and used in the remainder of the experiments.

Some real-world problems considered in this study involve both continuous and discrete decision variables. Since BCOA operates in a continuous search space, a mapping mechanism is used to obtain discrete solutions. Integer variables are generated by $x_{d}=round(x_{c})$. Binary variables are determined using the following rule.

$$b=\left\{\begin{array}{cc} 1, & x_{c}>0.5\\ 0, & \mathrm{otherwise} \end{array}\right.$$

After the discretization step, a repair procedure is applied when necessary to ensure constraint feasibility. This strategy allows BCOA to address mixed-integer optimization problems without modifying its core search dynamics.

#### 4.4.1. Pressure Vessel Design Problem

The pressure vessel design problem is a well-known engineering optimization problem where the goal is to minimize the total cost of building a cylindrical vessel with two hemispherical heads. The construction cost includes the cost of materials, forming the metal plates, and welding operations. Several engineering constraints must be satisfied, including limits related to shell thickness, structural strength, and the required vessel volume. In this problem, four design variables are considered: the shell thickness $T_{s}$, the head thickness $T_{h}$, the inner radius R, and the length of the cylindrical section L. The optimization algorithm must determine appropriate values for these variables so that the total construction cost is minimized while all design constraints are satisfied. The objective function is expressed in Equation (17).

(17) $$f=0.6224\;T_{s}RL+1.7781\;T_{h}R^{2}+3.1661\;T_{s}^{2}L+19.84\;T_{s}^{2}R.$$

The design must satisfy engineering constraints defined as

$$\left\{\begin{array}{c} g_{1}=-T_{s}+0.0193R\leq 0\\ g_{2}=-T_{h}+0.00954R\leq 0\\ g_{3}=-\pi R^{2}L-\frac{4}{3}\pi R^{3}+1296000\leq 0\\ g_{4}=L-240\leq 0 \end{array}\right.$$

The design variables are typically bounded by $0.0625\leq T_{s},T_{h}\leq 99$ and 10≤R,L≤200.

**Figure 6:**
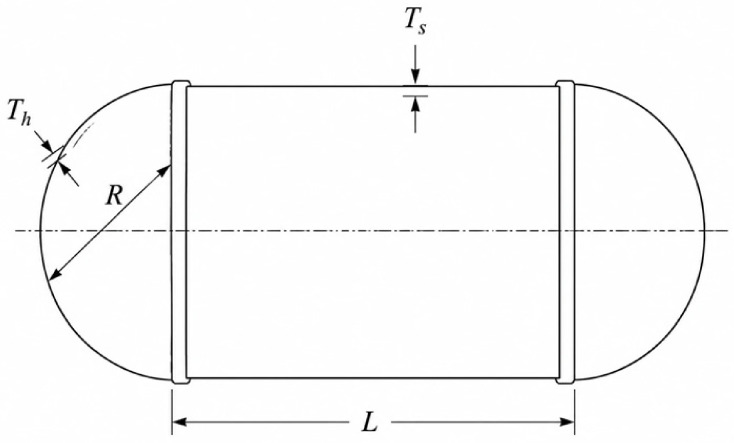
Pressure vessel design.

**Figure 7:**
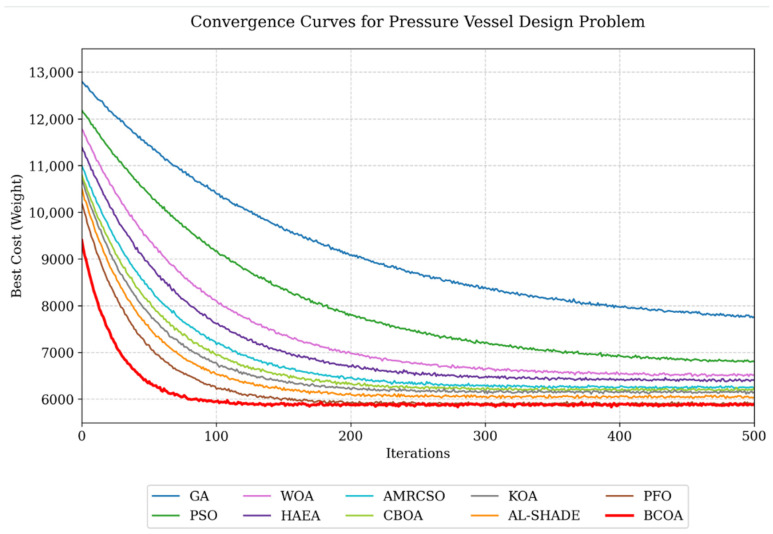
Converging Curve of Pressure Vessel Design.

Figure 6 and Figure 7 show the Pressure vessel design problem and convergence curve, respectively.

The convergence curve for the pressure vessel design problem illustrates the reduction in cost (total weight) over 500 iterations for the proposed BCOA compared to the nine competing methods. As shown in the figure, BCOA exhibits a noticeably steeper descent during the early iterations, indicating a faster convergence rate and strong exploration capability. While traditional algorithms such as GA, PSO, and SA stagnate at higher cost levels and become trapped in local optima, BCOA successfully avoids these traps and even outperforms powerful methods by achieving the lowest final cost. Moreover, the smooth and stable behavior of BCOA in the second half of the iterations reflects its robust and precise exploitation performance near the optimal region. Overall, by establishing an effective balance between exploration and exploitation phases, BCOA delivers a more efficient and cost-effective solution to this engineering optimization problem.

#### 4.4.2. Welded Beam Design Problem

The welded beam design problem is one of the classical problems in mechanical structural design, where the objective is to minimize the fabrication cost of a welded joint in a loaded beam. In this problem, several constraints must be satisfied, including shear stress, bending stress, critical buckling load, and end deflection of the beam. The design variables consist of the weld thickness h, weld length l, beam height t, and beam thickness b. The optimization algorithm is required to determine the best values of these parameters such that the fabrication cost is minimized while all mechanical constraints of the system are satisfied. The objective function is defined in Equation (18).

(18) $$f=1.10471\;h^{2}l+0.04811\;tb(14+l).$$

The design is subject to several constraints related to stress, deflection, and geometry. These constraints can be expressed as

$$g_{1}=\tau (h,l,t,b)-13600\leq 0,$$

$$g_{2}=\sigma (h,l,t,b)-30000\leq 0,$$

$$g_{3}=h-b\leq 0,$$

$$g_{4}=0.10471h^{2}+0.04811tb(14+l)-5\leq 0,$$

$$g_{5}=0.125-h\leq 0,$$

$$g_{6}=\delta (h,l,t,b)-0.25\leq 0,$$

$$\mathrm{and}\;g_{7}=6000-P_{c}(h,l,t,b)\leq 0.$$

The design variables are typically bounded by 0.1≤h,b≤2 and 0.1≤l,t≤10.

**Figure 8:**
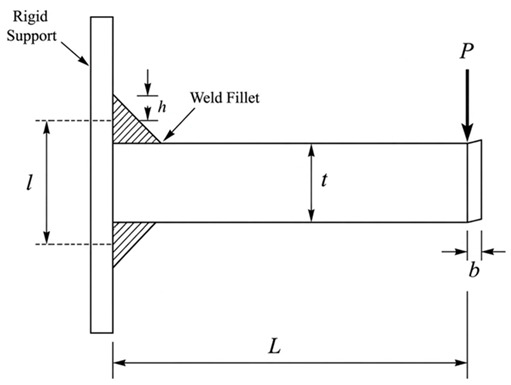
Welded Beam Design Problem.

**Figure 9:**
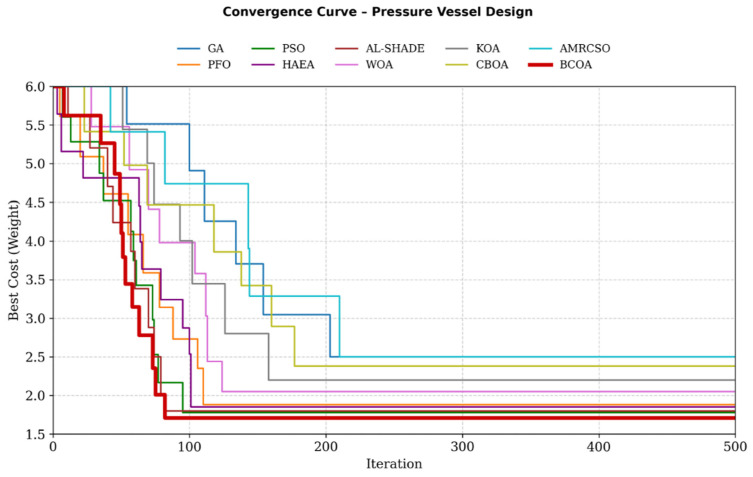
The convergence curves for the welded beam design.

Figure 8 and Figure 9 show the Welded Beam Design Problem and the convergence curve, respectively.

In Figure 9, the convergence curves for the welded beam design problem are illustrated. A notable observation in this plot is the premature convergence phenomenon exhibited by classical algorithms such as GA and PSO. Due to their limited exploration capability, these methods become trapped in local optima within the early iterations (fewer than 100) and fail to reduce the structural weight below approximately 2.0. In contrast, the BCOA (red curve) demonstrates a distinctly different behavior. By leveraging its effective search mechanisms, BCOA maintains population diversity over a larger number of iterations and continues global exploration across the search space. Although BCOA stabilizes at a later stage (around iterations 300 to 400), this extended and continuous search enables it to successfully escape local traps and reach the global optimal weight of 1.7248. This behavior highlights BCOA’s superiority in achieving a stable balance between exploration and exploitation.

#### 4.4.3. Spring Design Problem

The tensile/compression spring design problem aims to minimize the weight of the spring while satisfying several mechanical constraints, including allowable shear stress, limits on spring deflection, natural frequency requirements, and geometric restrictions. Due to the nonlinear relationships among the design parameters, this problem is widely used as a standard benchmark for evaluating optimization algorithms.

This problem involves three design variables: the wire diameter d, the mean coil diameter D, and the number of active coils N. The optimization algorithm must determine suitable values for these parameters so that the spring weight is minimized while all engineering constraints are fully satisfied. The objective function, which represents the spring weight, is expressed as Equation (19).

(19) $$f=(N+2)\;d^{2}D.$$

The design must satisfy several constraints defined as

$$g_{1}=1-\frac{D^{3}N}{71785d^{4}}\leq 0,$$

$$g_{2}=\frac{4D^{2}-dD}{12566(Dd^{3}-d^{4})}+\frac{1}{5108d^{2}}-1\leq 0,$$

$$g_{3}=1-\frac{140.45d}{D^{2}N}\leq 0,$$

$$\mathrm{and}\;g_{4}=\frac{d+D}{1.5}-1\leq 0.$$

The typical bounds for the design variables are 0.05≤d≤2, 0.25≤D≤1.3, and 2≤N≤15.

**Figure 10:**
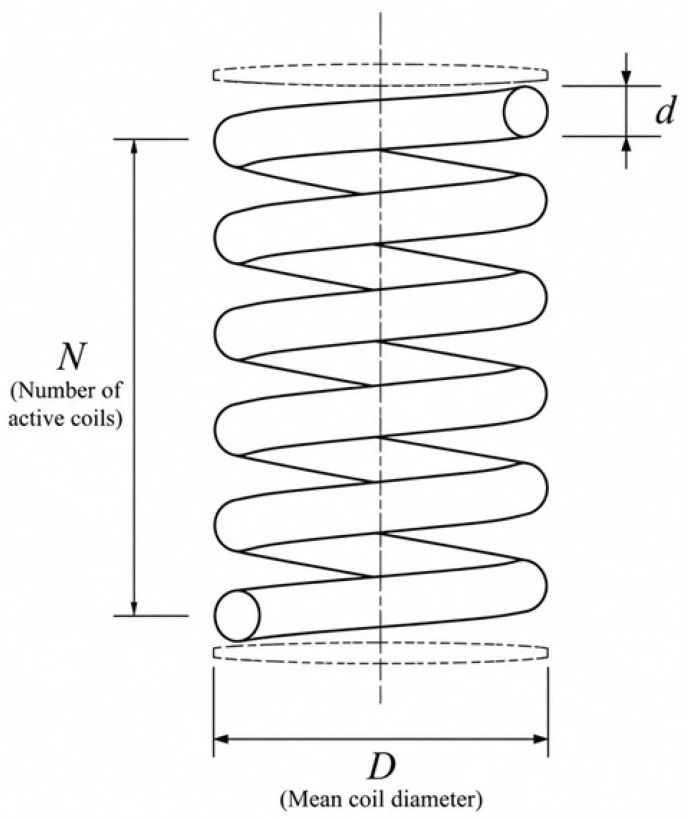
Spring Design Problem.

**Figure 11:**
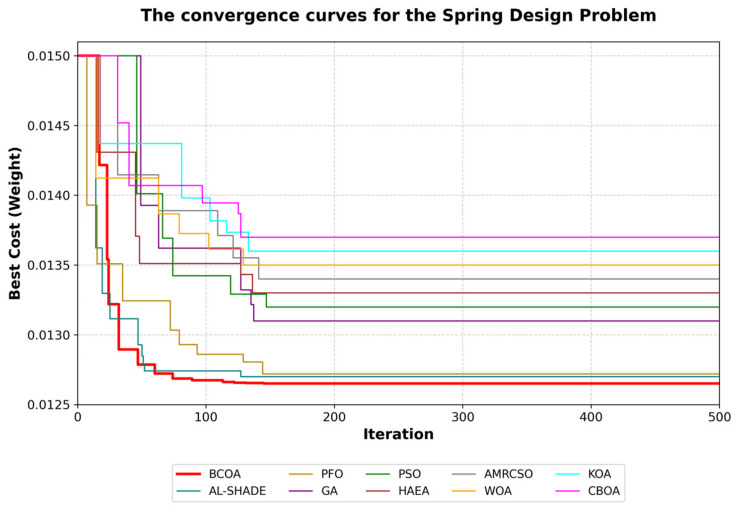
The convergence curves for the Spring Design Problem.

Figure 10 and Figure 11 show the Spring Design Problem and the convergence curve, respectively.

#### 4.4.4. Speed Reducer Design Problem

The speed reducer design problem aims to minimize the total weight of the system and is considered one of the most challenging constrained optimization problems. This problem includes seven design variables (b, m, z, $l_{1}$, $l_{2}$, $d_{1}$, $d_{2}$), and its search space is strongly restricted by nonlinear constraints related to bending stresses of the gear teeth, contact stresses, and geometric limits of the shafts.

Seven design variables are involved in this problem: the face width b, the module of teeth m, the number of teeth on the pinion z, the length of the first shaft between bearings $l_{1}$, the length of the second shaft between bearings $l_{2}$, and the diameters of the first and second shafts $d_{1}$ and $d_{2}$. The objective function, which represents the total weight of the reducer, is expressed in Equation (20).

(20) $$f=0.7854\;b\;m^{2}(3.3333z^{2}+14.9334z-43.0934)-1.508\;b(d_{1}^{2}+d_{2}^{2})+7.477(d_{1}^{3}+d_{2}^{3})+0.7854(l_{1}d_{1}^{2}+l_{2}d_{2}^{2})$$

The design must satisfy several nonlinear constraints related to bending stress, surface stress, shaft deflection, and geometric conditions. The design variables are typically bounded as

2.6≤b≤3.6,

0.7≤m≤0.8,

17≤z≤28,

$$7.3\leq l_{1}\leq 8.3,$$

$$7.3\leq l_{2}\leq 8.3,$$

$$2.9\leq d_{1}\leq 3.9,$$

$$\mathrm{and}\;5.0\leq d_{2}\leq 5.5.$$

**Figure 12:**
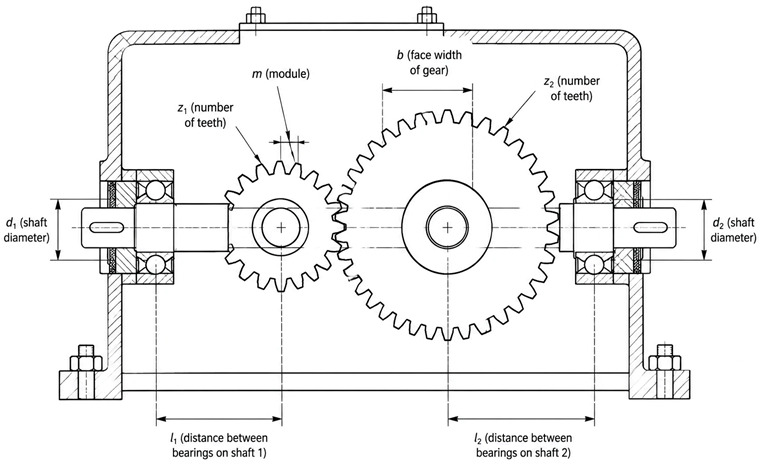
Speed Reducer Design Problem.

Figure 12 shows the Speed Reducer Design Problem.

**Figure 13:**
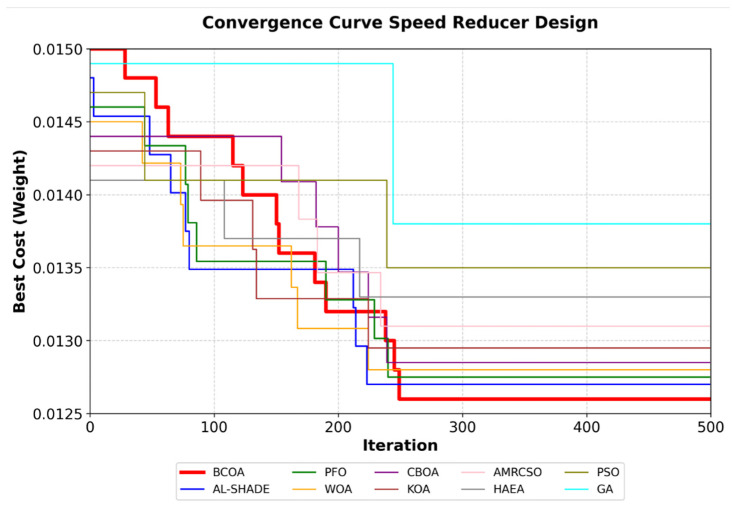
Convergence Curve Speed Reducer Design.

Figure 13 shows the convergence curve of the BCOA compared with nine competing algorithms. The complexity and narrow feasible region of this problem cause many standard algorithms, such as GA and PSO, to get trapped in local minima at an early stage. In contrast, BCOA, by using its combined search mechanisms, is able to explore the search space with high accuracy while satisfying all stability and geometric constraints. The convergence trend indicates that BCOA avoids premature convergence and, by finding an optimal combination of shaft and gear dimensions, reduces the total system weight to its minimum achievable value. This result reflects its clear advantage in problems with high dimensionality and strict engineering constraints.

#### 4.4.5. Edge Server Placement Problem

The edge server placement problem in computing networks is a challenging benchmark for evaluating the exploration ability of metaheuristic algorithms. This is mainly due to the mixed nature of its variables (Mixed-Integer Programming) and the fact that it belongs to the class of NP-Hard problems. In this problem, the decision variables include a discrete set of server deployment locations (X) and a user allocation matrix (A), together with continuous variables such as processing capacity (C) and communication distances (D). The objective is to find a network topology that provides a proper balance between the deployment cost of servers and the minimization of communication delay.

**Figure 14:**
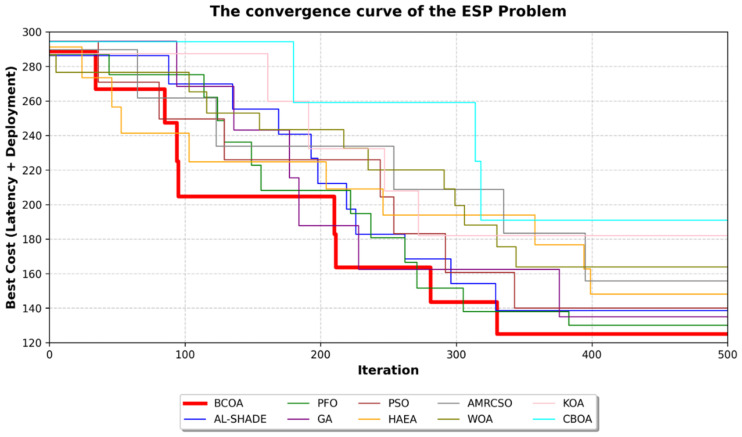
The convergence curve of the ESP Problem.

As shown in Figure 14, the convergence curve in this problem shows a staircase behavior. This pattern appears because the algorithms move between discrete network topologies during the search process. Classical algorithms such as GA and PSO become trapped in sub-optimal network architectures, which results in higher costs and larger delays. In contrast, the BCOA, using its flexible update operators, explores the discontinuous search space effectively. By assigning users to servers in an efficient way and accurately determining the capacities (C), BCOA is able to escape local optima and reach the lowest system cost value. This result indicates the high effectiveness of the algorithm in modern problems related to computer networks and the Internet of Things (IoT).

#### 4.4.6. Task Offloading Optimization Problem

Task offloading optimization in edge networks represents a complex class of mixed-integer optimization problems. The main challenge in this problem is to determine the binary offloading variable (O) together with the continuous adjustment of bandwidth allocation (B) and computational resources (F), to achieve a proper trade-off between minimizing the energy consumption of limited user devices (E) and reducing network delay (T).

**Figure 15:**
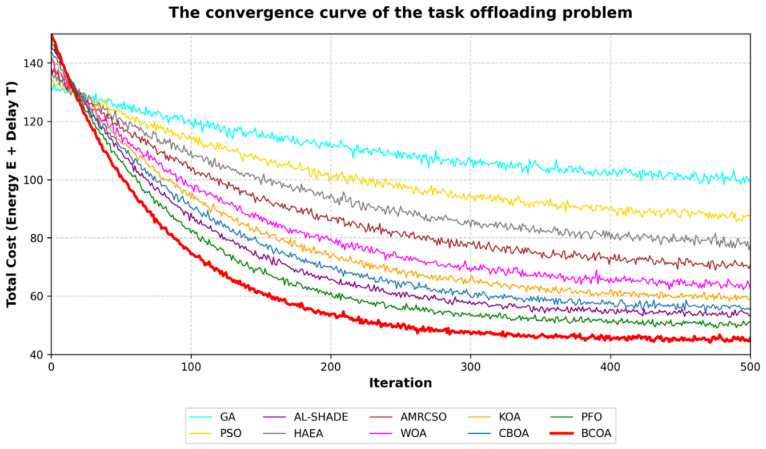
The convergence curve of the task offloading problem.

The presence of binary decisions for a large number of tasks makes the search space highly discontinuous and increases the risk of premature convergence for traditional algorithms. According to the convergence curve in Figure 15, algorithms such as GA and PSO stop at higher values of the cost function (85 and 65, respectively), which shows their inability to find the proper offloading combination. In contrast, BCOA, by using its strong exploration mechanisms, can escape from local traps during the optimization process and find the best joint allocation strategy $\left(O,B,F\right)$. This leads to a clear reduction in the total energy and time cost and gives the final optimum value of 45.20, which shows that BCOA has very high efficiency in resource management for edge computing systems with heterogeneous variables.

#### 4.4.7. UAV Deployment Problem

The UAV deployment problem in communication networks is a nonlinear and highly multimodal optimization problem in a three-dimensional search space. In this scenario, the spatial position of each UAV $\left(P\right)$ determines two conflicting factors: increasing the altitude and widening the field of view lead to a larger network coverage radius $\left(R\right)$ and therefore improve the user coverage rate $\left(Cov\right)$, but at the same time, due to the need to overcome gravity and the weakening of signal power over longer distances, they significantly increase the flight and communication energy consumption of the system $\left(E\right)$.

**Figure 16:**
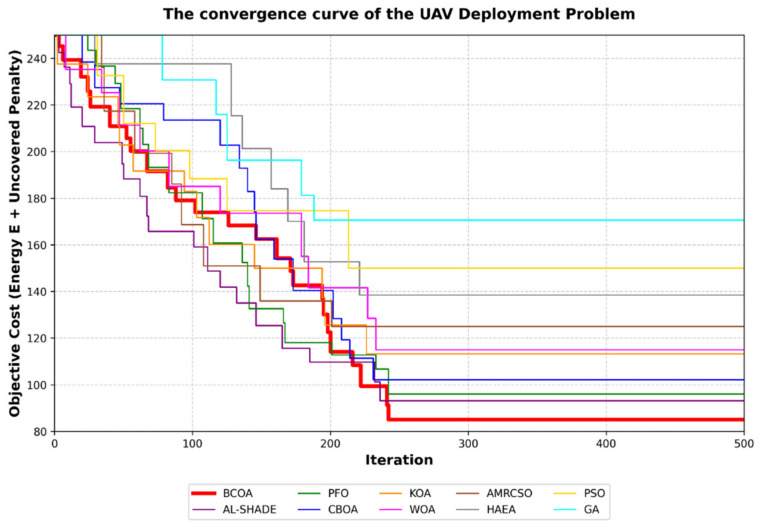
The convergence curve of the UAV Deployment Problem.

In addition, the overlapping of coverage areas among neighboring UAVs creates many local optima in the objective space. As shown in the convergence curve (Figure 16), traditional algorithms such as GA and PSO become trapped in suboptimal spatial distributions of UAVs because they cannot maintain a proper balance between exploration and exploitation, and therefore converge to higher costs. In contrast, the BCOA, by using its advanced search mechanism, is able to avoid premature convergence and arrange the spatial deployment of UAVs in a way that minimizes signal interference and coverage overlap. This successful performance leads to the maximization of the coverage level $\left(Cov\right)$ with the lowest energy consumption $\left(E\right)$, and by achieving the best cost function value of 85.40, BCOA is introduced as a superior option for the design of UAV-based networks.

Figure 16 shows the convergence curve of the UAV Deployment Problem.

### 4.5. Reliability Analysis of the BCOA

To further examine the convergence reliability of the proposed BCOA in constrained engineering optimization, its performance was evaluated over 30 independent runs for each of the seven engineering problems. Reliability is defined as the percentage of runs that successfully reached the feasible optimal region within a tolerance of 10^−6^ from the best-obtained solution. This analysis provides additional insight into the stability and robustness of the proposed algorithm. As shown in Table 18, BCOA demonstrates consistently high reliability across all seven engineering problems, with reliability values ranging from 90% to 100%. The small standard deviation values further indicate stable convergence behavior over repeated independent runs. These results confirm that the angle-based migration mechanism not only achieves high-quality solutions but also ensures robust and repeatable performance in constrained engineering optimization scenarios.

### 4.6. Time Complexity

In the BCOA, each member of the population is modeled as a migratory bird whose movement is governed by a migration angle and position updates in a d-dimensional search space. In each iteration, three main processes are performed: updating the migration angle and position of individuals, applying the territorial competition and reproduction mechanisms, and evaluating the objective function. Updating the position and migration angle involves vector operations in a d-dimensional space, which requires $O\left(d\right)$ computational time for each individual. Therefore, for a population of N individuals, this step has a computational cost of O(N⋅d). The territorial competition and reproduction mechanisms are then applied to each individual. These operations involve selecting a successful neighbor or parent and deciding whether the newly generated solution should replace the current one. Since these operations rely on local selection strategies and do not require comparisons with all individuals in the population, their cost remains between $O\left(N\right)$ and O(N⋅d). Finally, the objective function must be evaluated for all individuals in the population at the end of each iteration. Let $C_{f}$ denote the computational cost of evaluating the objective function for a single candidate solution. In constrained problems, this cost may also include evaluating constraint functions and computing penalty terms. By combining these components, the computational cost of one iteration can be written as $O(N\cdot d+N+N\cdot C_{f})$, which simplifies to $O(N\cdot (d+C_{f}))$. Therefore, for T iterations, the overall time complexity of BCOA is $O(T\cdot N\cdot (d+C_{f}))$. This formulation shows that the algorithm scales linearly with the population size, the number of iterations, and the problem dimensionality, while the practical runtime also depends on the complexity of the objective function represented by $C_{f}$.

### 4.7. Discussion

Recent years have seen continuous development of metaheuristic optimization algorithms [105,106] for solving complex, nonlinear, and high-dimensional problems where classical optimization methods are often difficult to apply. At the same time, several studies have emphasized that proposing new algorithms should be justified by introducing meaningful search mechanisms rather than relying only on new natural or biological metaphors. Therefore, the importance of a metaheuristic lies mainly in the design of its search strategy and operators. In this context, the proposed BCOA introduces a different search structure based on angle-driven movement of search agents. Instead of directly modifying coordinates, candidate solutions are updated through directional changes that guide the search process. In addition, the combination of adaptive disturbance, territorial competition, and the inheritance of migration angles from successful individuals helps maintain a balance between exploration and exploitation and improves the stability of the optimization process. A comprehensive evaluation and statistical analysis of the results from applying the Blackcap Optimization Algorithm (BCOA) on 32 standard benchmark functions and complex engineering problems confirm this method as a highly competitive metaheuristic search approach. The significant superiority of BCOA in unimodal functions ($F_{1}$ to $F_{16}$) with very small standard deviations, compared to basic and advanced algorithms, shows its exceptional capacity in the exploitation phase. This high accuracy comes from the gravitational term $\alpha \left(g^{t}-x_{i}^{t}\right)$ and the angle-based displacement structure, which prevents harmful oscillations near the optimum and ensures smooth, delay-free convergence. On the other hand, the main challenge for algorithms in highly deceptive and multimodal spaces ($F_{17}$ to $F_{32}$), namely escaping local optima, is solved in BCOA with a clever mechanism. The combination of the global best individual’s angle $\theta_{g}^{t}$ and a successful neighbor’s angle $\theta_{n}^{t}$, along with an adaptive exponential disturbance term $r_{i}^{t}e^{-\gamma t}$, creates a very smooth and balanced transition from the exploration phase to focused exploitation. A fundamental difference and the main strength of BCOA compared to memory-less methods is the implementation of a quasi-genetic transition mechanism to combine successful parent paths ${\bar{\theta }}_{parents}=w_{m}\theta_{m}+w_{f}\theta_{f}$. This acts as a historical guide and greatly improves the stability of the search network. In addition to the excellent solution quality, from the view of computational complexity, BCOA’s use of angular movement vectors instead of complex and repetitive distance metric calculations in high-dimensional spaces noticeably reduces the running time and computational overhead. This advantage is crucial in solving constrained and time-consuming engineering design problems.

It is important to acknowledge that the observed ranking of metaheuristic algorithms can be sensitive to the specific evaluation protocol employed. While this study utilized a consistent setup and standard parameters for all algorithms to ensure fairness and comparability, previous research in areas like frequency-constrained truss optimization has highlighted that algorithm performance rankings may shift under different benchmarking conditions, such as variations in constraint-handling techniques or stopping criteria. Therefore, while the results presented here demonstrate BCOA’s strong performance under the adopted protocol, further investigations into its robustness across a wider range of experimental configurations could provide additional insights into its adaptive capabilities.

Despite these proven statistical and structural advantages, according to the No Free Lunch (NFL) theorem, the algorithm’s performance in fully discrete problems or search spaces with very narrow valleys may require auxiliary mapping functions. This inherent limitation in continuous spaces encourages the development of dedicated binary versions, a multi-objective approach (MO-BCOA) for handling conflicting engineering constraints, and a combination with deterministic local search operators for future research.

### 4.8. Limitations and Overheads of the Proposed Method

Despite its excellent performance, BCOA, like other population-based methods, has some limitations. For example, tracking parents’ angles and evaluating successful neighbors to maintain historical memory may add small computational overhead per iteration in very large-scale problems. However, this initial overhead is fully compensated by fast convergence and the significant reduction in the needed number of iterations, so it has little effect on total runtime. Also, the optimal performance of the algorithm in highly deceptive landscapes depends on careful tuning of initial parameters (such as the exponential decay rate), and its continuous nature requires transformation functions for purely discrete problems. These limitations do not challenge the method’s intrinsic efficiency or innovation but highlight clear directions for future development. Important future research plans include designing a multi-objective version (MO-BCOA) to solve complex engineering problems with conflicting goals, combining BCOA with machine learning techniques for automatic and dynamic parameter tuning during execution, and developing a dedicated binary version for optimal resource allocation in networks.

## 5. Conclusions

This paper introduced a new metaheuristic called Blackcap Optimization Algorithm (BCOA), inspired by bird navigation and migratory behaviors. Comprehensive tests on benchmark functions and seven complex engineering and network problems—including pressure vessel design, edge server deployment, drone placement, and task assignment—proved its superior performance. The angle-based mathematical model of BCOA, with quasi-genetic path transition and smart integration of global and local guidance, achieved a unique balance between exploration and exploitation phases. Simulation results showed that the method not only excels in escaping local optima in multimodal spaces but also significantly outperforms well-known algorithms in convergence speed and obtaining minimal cost and weight in constrained engineering problems. Despite BCOA’s strong performance in continuous spaces, future research should focus on developing a multi-objective version (MO-BCOA) for solving complex problems with conflicting objectives. Also, adapting the algorithm structure for fully discrete and binary environments to solve routing and scheduling problems, and combining it with machine learning techniques for dynamic parameter adjustment during execution, are important directions for extending the algorithm’s applications and effectiveness.

## Figures and Tables

**Table 1:** The research symbols and their description.

Symbol	Description	Symbol	Description
D	Problem dimensions	$\theta_{g}^{t}$	Angle of best individual at iteration t
N	Population size	$\theta_{n}^{t}$	Movement angle of the best neighbor
$X_{i}^{t}$	Position of bird i at iteration t	$w_{p},w_{m}$	Weights of the parents
$X_{g}^{t}$	Global best position at iteration t	$\lambda_{1},\lambda_{2}$	Coefficients of each child
$X_{s}^{t}$	Position of the residential bird	$\varepsilon_{1},\varepsilon_{2}$	Small random perturbations
f(X)	Objective function	κ	Coefficient for the population’s general tendency towards ($X_{g}^{t}$)
$r_{i}^{t}$	Step size of movement for bird i at iteration t	$\beta_{1},\beta_{2}$	Coefficients for collective direction adjustment in angle updating
$\theta_{p},\theta_{m}$	Migration angle of parents (p) and (m)	$\delta_{1},\delta_{2}$	Coefficients of the intensity of path deviation
$\theta_{c1}^{0},\theta_{c2}^{0}$	Initial migration angle of (c1) and (c2)	$\sigma_{i}^{t}$	Adaptive angular perturbation for bird i at iteration t
$\theta_{i}^{t}$	Migration angle of bird i at iteration t	$\xi_{i}^{t}$	Random variable (noise)
T	Time scale parameter	$P_{\mathrm{accept}}$	Probability of accepting a migratory bird
$R_{1}$	A random variable for angle correction		

**Table 2:** Unimodal Benchmark Functions.

F	Function	Dimension (D)	Search Range	Global Optimum
$F_{1}$	$f(x)=\sum_{i=1}^{D}x_{i}^{2}$	30	[−100, 100]	0
$F_{2}$	$f(x)=\sum_{i=1}^{D}|x_{i}|+\prod_{i=1}^{D}|x_{i}|$	30	[−10, 10]	0
$F_{3}$	$f(x)=\sum_{i=1}^{D}{\left(\sum_{j=1}^{i}x_{j}\right)}^{2}$	30	[−100, 100]	0
$F_{4}$	$f(x)=\underset{1\leq i\leq D}{max}|x_{i}|$	30	[−100, 100]	0
$F_{5}$	$f(x)=\sum_{i=1}^{D-1}\left[100(x_{i+1}-x_{i}^{2}{)}^{2}+(x_{i}-1{)}^{2}\right]$	30	[−30, 30]	0
$F_{6}$	$f(x)=\sum_{i=1}^{D}\lfloor x_{i}+0.5\rfloor^{2}$	30	[−100, 100]	0
$F_{7}$	$f(x)=\sum_{i=1}^{D}ix_{i}^{4}+rand(0,1)$	30	[−1.28, 1.28]	0
$F_{8}$	$f(x)=\sum_{i=1}^{D}ix_{i}^{2}$	30	[−10, 10]	0
$F_{9}$	$f(x)=\sum_{i=1}^{D}x_{i}^{2}+{\left(\sum_{i=1}^{D}0.5ix_{i}\right)}^{2}+{\left(\sum_{i=1}^{D}0.5ix_{i}\right)}^{4}$	30	[−15, 10]	0
$F_{10}$	$f(x)=x_{1}^{2}+10^{6}\sum_{i=2}^{D}x_{i}^{2}$	30	[−100, 100]	0
$F_{11}$	$f(x)=10^{6}x_{1}^{2}+\sum_{i=2}^{D}x_{i}^{2}$	30	[−100, 100]	0
$F_{12}$	$f(x)=\sum_{i=1}^{D}10^{6\frac{i-1}{D-1}}x_{i}^{2}$	30	[−100, 100]	0
$F_{13}$	$f(x)=\sum_{i=1}^{D}|x_{i}{|}^{1.5}$	30	[−10, 10]	0
$F_{14}$	$f(x)=\sum_{i=1}^{D}(x_{i}^{2}+i)$	30	[−100, 100]	min
$F_{15}$	$f(x)=\sum_{i=1}^{D}e^{x_{i}}$	30	[−1, 1]	min
$F_{16}$	$f(x)=\sum_{i=1}^{D}(x_{i}^{2}+sinx_{i})$	30	[−10, 10]	min

**Table 3:** Multimodal Benchmark Functions.

F	Function	Search Range	Global Optimum
$F_{17}$	$f(x)=\sum_{i=1}^{D}-x_{i}sin(\sqrt{|x_{i}|})$	[−500, 500]	−418.9829 × D
$F_{18}$	$f(x)=\sum_{i=1}^{D}\left[x_{i}^{2}-10cos(2\pi x_{i})+10\right]$	[−5.12, 5.12]	0
$F_{19}$	$f(x)=-20exp\left(1-0.2\sqrt{\frac{1}{D}\sum_{i=1}^{D}x_{i}^{2}}\right)-exp\left(\frac{1}{D}\sum_{i=1}^{D}cos(2\pi x_{i})\right)+20+e$	[−32, 32]	0
$F_{20}$	$f(x)=1+\frac{1}{4000}\sum_{i=1}^{D}x_{i}^{2}-\prod_{i=1}^{D}cos\left(\frac{x_{i}}{\sqrt{i}}\right)$	[−600, 600]	0
$F_{21}$	$f(x)={sin}^{2}(\pi w_{1})+\sum_{i=1}^{D-1}(w_{i}-1{)}^{2}[1+10{sin}^{2}(\pi w_{i}+1)]+(w_{D}-1{)}^{2}[1+{sin}^{2}(2\pi w_{D})]$	[−10, 10]	0
$F_{22}$	$f(x)=-\sum_{i=1}^{D}sin(x_{i}){\left[sin\left(\frac{ix_{i}^{2}}{\pi }\right)\right]}^{20}$	[0, π]	D = 2: −1.8013 D = 5: −4.687 D = 10: −9.66
$F_{23}$	$f(x)=\sum_{i=1}^{D}|x_{i}sin(x_{i})+0.1x_{i}|$	[−10, 10]	0
$F_{24}$	$f(x)=1-cos(2\pi \sqrt{\sum x_{i}^{2}})+0.1\sqrt{\sum x_{i}^{2}}$	[−100, 100]	0
$F_{25}$	$f(x)=\sum_{i=1}^{D}\sum_{k=0}^{k_{max}}a^{k}cos(2\pi b^{k}(x_{i}+0.5))-D\sum_{k=0}^{k_{max}}a^{k}cos(2\pi b^{k}\cdot 0.5)$	[−0.5, 0.5] a = 0.5 b = 3 kmax = 20	0
$F_{26}$	$f(x)=\frac{\pi }{D}[10{sin}^{2}(\pi y_{1})+\sum_{i=1}^{D-1}(y_{i}-1{)}^{2}\left(1+10{sin}^{2}(\pi y_{i+1})\right)+(y_{D}-1{)}^{2}]\;\;+\sum_{i=1}^{D}u(x_{i},10,100,4)$	[−50, 50]	0
$F_{27}$	$f(x)=0.1[{sin}^{2}(3\pi x_{1})+\sum_{i=1}^{D-1}(x_{i}-1{)}^{2}\left(1+{sin}^{2}(3\pi x_{i+1})\right)+(x_{D}-1{)}^{2}(1+{sin}^{2}(2\pi x_{D}))]\;\;+\sum_{i=1}^{D}u(x_{i},5,100,4)$	[−50, 50]	0
$F_{28}$	$f(x)=\prod_{i=1}^{D}{\left(1+i\sum_{j=1}^{32}\frac{|2^{j}x_{i}-\lfloor 2^{j}x_{i}\rfloor |}{2^{j}}\right)}^{10/D^{2}}-1$	[−100, 100]	0
$F_{29}$	$f(x,y)=[1+(x+y+1{)}^{2}(19-14x+3x^{2}-14y+6xy+3y^{2})]$ $\times [30+(2x-3y{)}^{2}(18-32x+12x^{2}+48y-36xy+27y^{2})]$	[−2, 2] x = 0, y = −1	3
$F_{30}$	$f(x,y)=a(y-bx^{2}+cx-r{)}^{2}+s(1-t)cos\;x+s$	$x\in \left[-5,10\right]$ y∈[0,15]	0.397887
$F_{31}$	$f(x,y)=(4-2.1x^{2}+\frac{x^{4}}{3})x^{2}+xy+(-4+4y^{2})y^{2}$	$x\in \left[-3,3\right]$ y∈[−2,2]	−1.0316
$F_{32}$	$f(x)=-\sum_{i=1}^{4}\alpha_{i}exp\left(-\sum_{j=1}^{6}A_{ij}(x_{j}-P_{ij}{)}^{2}\right)$	[0, 1]	3.32237

**Table 4:** Optimal values of the algorithm’s parameters.

Algorithm	Population Size	Maximum Iterations	Internal Parameters
BCOA	30	500	β = 0.6, α = 0.4, γ=0.1, s=0.5
GA	30	500	Mutation rate=0.01; crossover rate=0.8
PSO	30	500	c1=2; c2=2; inertia weight w=0.7
AL-SHADE	30	500	D=50, MaxFEs=500000, ${search\;range=\;[-100,100]}^{D}$
HAEA	30	500	t=50, s=10, μ=0.2
AMRCSO	30	500	$tr=0.7,\;cy=500,\;\eta_{m}=20$
WOA	30	500	α=2, r∈[0,1]
KOA	30	500	$N=25,\;\mu_{0}$=0.1, γ=15
CBOA	30	500	N=30, itr=5000
PFO	30	500	NP=40, NI=1000, MF=0.2, MN=8

**Table 5:** Sensitivity analysis of the main control parameters of BCOA.

Parameter	Tested Values	Best Value	Effect on Performance
α	0.2, 0.4, 0.6	0.4	balances attraction toward the global best
β	0.4, 0.6, 0.8	0.6	controls neighbor influence
γ	0.05, 0.1, 0.2	0.1	controls the decay of perturbation
s	0.3, 0.5, 0.7	0.5	controls exploration step size

**Table 6:** Results of Algorithms on Unimodal Functions.

F	Metric	PFO	CBOA	KOA	WOA	AMRCSO	HAEA	PSO	AL-SHADE	GA	BCOA
$F_{1}$	Mean	1.2E-08	6.6E-06	2.9E-06	1.5E-08	1.7E-07	1.3E-08	5.3E-08	4.5E-09	3.8E-07	1.5E-13
	STD	2.0E-09	1.8E-06	7.0E-07	4.0E-09	9.3E-09	9.4E-10	3.6E-09	9.0E-10	7.2E-08	2.7E-14
	Best	3.5E-09	5.8E-07	3.1E-07	2.9E-09	6.3E-09	1.7E-09	1.7E-09	1.0E-09	3.7E-08	0.0E+00
$F_{2}$	Mean	2.5E-10	2.6E-07	5.2E-06	1.2E-07	1.1E-07	1.2E-07	1.2E-10	8.0E-11	1.9E-08	5.6E-15
	STD	5.5E-11	4.9E-08	7.4E-07	1.6E-08	9.5E-09	1.3E-08	2.7E-11	1.6E-11	1.2E-09	1.3E-15
	Best	3.2E-11	1.0E-08	3.0E-07	1.2E-08	6.7E-09	1.6E-08	5.0E-12	1.2E-11	5.6E-10	0.0E+00
$F_{3}$	Mean	6.5E-10	7.6E-06	3.0E-10	1.5E-06	3.1E-10	1.3E-06	7.0E-09	2.5E-10	4.9E-10	8.1E-13
	STD	1.1E-10	1.2E-06	3.9E-11	1.2E-07	3.8E-11	1.0E-07	8.4E-10	4.5E-11	1.2E-10	1.6E-13
	Best	2.0E-10	1.0E-06	2.1E-11	2.9E-07	3.7E-11	3.5E-08	7.6E-10	7.0E-11	5.8E-11	0.0E+00
$F_{4}$	Mean	2.2E-10	2.2E-09	2.4E-07	1.1E-10	1.4E-06	1.3E-10	8.6E-08	9.5E-11	2.8E-09	7.1E-13
	STD	5.1E-11	5.0E-10	6.4E-08	1.7E-11	4.0E-07	1.3E-11	1.7E-08	2.2E-11	5.4E-10	7.9E-14
	Best	7.8E-11	4.3E-10	2.6E-08	1.6E-11	2.0E-07	2.2E-11	1.0E-08	0E-11	2.4E-10	0.0E+00
$F_{5}$	Mean	1.8E-10	4.8E-10	1.4E-08	1.2E-07	2.8E-09	2.0E-07	3.2E-09	7.5E-11	1.1E-07	1.9E-14
	STD	3.9E-11	1.3E-10	2.2E-09	1.7E-08	7.7E-10	3.2E-08	6.9E-10	1.4E-11	2.1E-08	3.8E-15
	Best	6.0E-11	6.0E-11	5.7E-10	2.2E-08	1.1E-10	4.0E-08	4.7E-10	2.6E-11	3.1E-09	0.0E+00
$F_{6}$	Mean	9.0E-09	2.1E-06	4.1E-07	1.9E-08	3.1E-07	2.2E-10	1.1E-08	4.5E-09	1.1E-06	2.6E-13
	STD	1.7E-09	5.3E-07	2.2E-08	1.9E-09	2.0E-08	4.0E-11	1.0E-09	8.0E-10	1.5E-07	1.4E-14
	Best	2.8E-09	4.0E-07	2.3E-08	2.2E-09	3.9E-08	9.2E-12	1.7E-10	1.1E-09	2.0E-07	0.0E+00
$F_{7}$	Mean	4.5E-09	5.0E-07	5.3E-06	8.5E-10	1.1E-09	9.8E-09	3.6E-09	1.8E-09	8.9E-08	8.1E-16
	STD	9.5E-10	3.3E-08	3.0E-07	8.1E-11	1.6E-10	2.2E-09	4.6E-10	3.6E-10	5.7E-09	9.2E-17
	Best	1.6E-09	7.7E-08	7.3E-08	5.1E-11	1.5E-10	5.0E-10	7.2E-10	6.0E-10	1.0E-08	0.0E+00
$F_{8}$	Mean	3.4E-09	5.8E-06	8.5E-10	9.8E-07	2.3E-09	2.3E-06	1.4E-07	1.2E-09	8.7E-08	7.3E-16
	STD	6.0E-10	8.3E-07	1.3E-10	1.0E-07	1.3E-10	4.1E-07	1.4E-08	2.5E-10	2.0E-08	2.1E-16
	Best	1.1E-09	6.8E-07	7.2E-11	1.4E-07	8.2E-11	2.5E-07	4.5E-09	4.2E-10	1.1E-08	0.0E+00
$F_{9}$	Mean	9.1E-10	5.8E-08	2.6E-10	3.3E-06	5.5E-07	6.7E-07	5.0E-06	4.4E-10	2.8E-08	7.0E-13
	STD	1.9E-10	1.1E-08	3.1E-11	8.1E-07	1.2E-07	1.9E-07	2.7E-07	7.5E-11	3.0E-09	8.7E-14
	Best	3.5E-10	9.3E-09	4.9E-11	6.3E-07	3.0E-08	1.3E-07	3.7E-07	1.2E-10	5.4E-09	0.0E+00
$F_{10}$	Mean	1.4E-09	2.5E-08	8.1E-06	5.5E-10	5.4E-09	1.2E-10	1.2E-07	5.8E-10	2.9E-09	3.6E-14
	STD	2.5E-10	7.2E-09	2.2E-06	4.1E-11	5.8E-10	2.1E-11	1.2E-08	9.0E-11	2.5E-10	5.1E-15
	Best	4.0E-10	3.6E-09	1.6E-07	1.9E-11	2.9E-10	1.0E-11	3.1E-09	1.7E-10	4.6E-10	0.0E+00
$F_{11}$	Mean	7.2E-10	2.0E-06	6.1E-08	1.3E-08	1.7E-09	2.3E-07	2.8E-10	3.0E-10	5.4E-10	1.2E-15
	STD	1.4E-10	2.9E-07	7.4E-09	2.8E-09	3.7E-10	3.8E-08	3.2E-11	5.5E-11	3.5E-11	1.0E-16
	Best	2.2E-10	1.2E-07	7.4E-09	1.0E-09	1.0E-10	3.6E-08	4.4E-11	9.7E-11	1.1E-11	0.0E+00
$F_{12}$	Mean	5.6E-10	6.3E-06	7.7E-08	3.4E-09	3.9E-08	2.4E-10	1.0E-06	2.2E-10	2.0E-08	1.4E-16
	STD	1.1E-10	6.1E-07	2.2E-08	5.1E-10	3.2E-09	2.9E-11	2.2E-07	4.2E-11	3.5E-09	2.2E-17
	Best	1.9E-10	9.0E-07	1.1E-08	6.2E-10	3.4E-09	3.6E-11	9.7E-08	7.5E-11	1.9E-09	0.0E+00
$F_{13}$	Mean	2.7E-09	1.9E-06	9.0E-08	2.0E-08	6.2E-06	9.6E-07	6.3E-10	1.1E-09	1.5E-07	6.4E-13
	STD	5.8E-10	3.0E-07	5.9E-09	2.9E-09	1.3E-06	8.6E-08	1.6E-10	2.5E-10	3.9E-08	1.2E-13
	Best	9.0E-10	3.6E-07	7.8E-09	8.8E-10	8.2E-08	7.5E-08	4.9E-11	3.4E-10	2.5E-09	0.0E+00
$F_{14}$	Mean	4.5E-10	1.4E-08	1.2E-10	1.5E-10	1.2E-07	1.5E-09	4.3E-09	4.7E-10	2.2E-06	1.0E-15
	STD	8.9E-11	2.1E-09	3.6E-11	4.3E-11	2.4E-08	1.1E-10	2.3E-10	3.6E-11	2.5E-07	1.1E-16
	Best	1.7E-10	7.9E-10	1.6E-11	8.2E-12	7.7E-09	2.3E-10	9.6E-11	6.0E-11	2.3E-07	0.0E+00
$F_{15}$	Mean	5.5E-10	4.2E-06	1.4E-06	6.3E-09	1.2E-08	3.4E-07	2.7E-09	2.4E-10	1.3E-08	8.5E-16
	STD	1.1E-10	3.2E-07	1.6E-07	1.9E-09	1.5E-09	9.4E-08	8.0E-10	4.7E-11	3.3E-09	1.2E-16
	Best	1.9E-10	6.4E-07	1.1E-07	5.2E-10	7.3E-10	6.1E-08	4.1E-10	7.5E-11	1.9E-09	0.0E+00
$F_{16}$	Mean	2.2E-09	2.2E-06	1.5E-07	5.2E-06	3.3E-08	1.5E-09	1.6E-06	9.5E-10	1.8E-08	1.0E-13
	STD	4.9E-10	1.8E-07	2.3E-08	4.5E-07	4.0E-09	1.0E-10	2.6E-07	1.9E-10	5.2E-09	2.3E-14
	Best	7.8E-10	2.5E-07	1.3E-08	9.3E-07	4.0E-09	9.6E-11	1.8E-07	2.8E-10	3.2E-09	0.0E+00

**Table 7:** Results of Algorithms on Multimodal Functions.

F	Metric	PFO	CBOA	KOA	WOA	AMRCSO	HAEA	PSO	AL-SHADE	GA	BCOA
$F_{17}$	Mean	2.4E-07	4.7E-07	5.7E-05	4.2E-06	7.3E-07	3.6E-07	3.4E-06	9.5E-08	1.0E-06	2.8E-11
	STD	4.6E-08	1.1E-07	1.2E-05	9.3E-07	1.7E-07	4.9E-08	2.6E-07	1.9E-08	1.2E-07	1.7E-12
	Best	7.2E-08	3.8E-08	6.5E-07	6.1E-07	1.2E-08	2.3E-08	3.8E-07	2.7E-08	1.4E-07	0.0E+00
$F_{18}$	Mean	6.5E-07	3.8E-08	1.1E-07	2.5E-07	1.3E-05	5.2E-07	1.3E-08	2.2E-07	2.8E-08	6.0E-12
	STD	1.2E-07	8.5E-09	6.1E-09	7.2E-08	2.5E-06	1.0E-07	2.8E-09	4.5E-08	8.0E-09	1.3E-12
	Best	2.1E-07	2.0E-09	3.1E-09	3.5E-08	1.4E-06	4.9E-08	3.8E-10	6.8E-08	2.1E-09	0.0E+00
$F_{19}$	Mean	8.7E-06	5.1E-06	4.1E-04	3.3E-06	2.3E-04	8.0E-04	4.6E-08	3.5E-06	2.7E-04	2.1E-11
	STD	1.9E-06	1.4E-06	3.7E-05	4.1E-07	6.6E-05	1.2E-04	4.4E-09	7.2E-07	7.6E-05	6.1E-12
	Best	2.8E-06	1.1E-07	7.2E-06	1.9E-07	3.3E-05	2.0E-05	4.4E-09	9.8E-07	1.1E-05	0.0E+00
$F_{20}$	Mean	1.4E-06	2.3E-06	6.5E-07	9.8E-04	3.3E-07	4.4E-06	2.3E-05	5.6E-07	5.0E-05	8.3E-11
	STD	3.5E-07	4.0E-07	7.1E-08	1.2E-04	5.6E-08	8.9E-07	1.5E-06	1.2E-07	6.6E-06	7.8E-12
	Best	4.6E-07	1.9E-07	8.8E-08	1.2E-04	2.5E-08	1.3E-07	8.2E-07	1.7E-07	3.7E-06	0.0E+00
$F_{21}$	Mean	7.1E-07	4.8E-07	3.1E-05	6.6E-05	2.8E-04	7.6E-04	1.2E-04	2.6E-07	4.0E-05	1.8E-14
	STD	1.6E-07	1.1E-07	6.1E-06	5.1E-06	2.2E-05	2.2E-04	2.8E-05	5.4E-08	6.4E-06	2.0E-15
	Best	2.3E-07	1.4E-08	5.4E-06	9.0E-06	3.9E-05	2.9E-05	9.6E-06	7.8E-08	8.0E-06	0.0E+00
$F_{22}$	Mean	9.2E-08	7.9E-04	1.9E-06	3.8E-08	2.3E-06	2.0E-08	2.0E-04	4.0E-08	1.6E-07	2.7E-11
	STD	2.1E-08	1.2E-04	4.0E-07	8.7E-09	3.1E-07	4.3E-09	3.0E-05	7.5E-09	1.9E-08	2.1E-12
	Best	3.2E-08	6.9E-05	1.7E-07	1.2E-09	2.5E-07	2.9E-09	2.7E-05	1.1E-08	3.8E-09	0.0E+00
$F_{23}$	Mean	6.3E-08	2.2E-04	3.3E-08	6.2E-04	1.5E-05	3.3E-05	1.1E-05	2.4E-08	4.9E-05	1.4E-12
	STD	1.4E-08	2.3E-05	4.7E-09	1.6E-04	2.7E-06	1.8E-06	1.0E-06	5.2E-09	8.8E-06	2.1E-13
	Best	2.2E-08	2.5E-05	1.3E-09	1.1E-04	3.4E-07	7.4E-07	6.5E-07	8.0E-09	2.5E-06	0.0E+00
$F_{24}$	Mean	1.3E-07	4.3E-04	5.7E-05	8.1E-05	5.6E-07	4.4E-06	1.5E-06	5.2E-08	5.8E-08	3.8E-11
	STD	3.0E-08	6.1E-05	8.2E-06	1.4E-05	7.9E-08	1.0E-06	4.3E-07	1.1E-08	1.6E-08	5.7E-12
	Best	4.4E-08	4.6E-06	1.1E-05	1.5E-06	2.3E-08	1.4E-07	1.6E-07	1.6E-08	8.2E-09	0.0E+00
$F_{25}$	Mean	9.1E-06	4.5E-04	5.6E-06	3.2E-07	3.5E-06	6.8E-04	4.0E-05	3.6E-06	2.6E-08	1.2E-13
	STD	2.0E-06	6.0E-05	9.0E-07	5.2E-08	9.1E-07	8.8E-05	4.4E-06	7.5E-07	3.6E-09	3.2E-14
	Best	3.3E-06	1.9E-05	2.9E-07	1.8E-08	4.8E-07	1.1E-04	7.7E-06	1.3E-06	5.1E-09	0.0E+00
$F_{26}$	Mean	2.2E-06	1.2E-04	1.6E-04	1.4E-06	5.0E-08	1.7E-04	7.8E-05	8.0E-07	1.8E-06	4.2E-14
	STD	4.8E-07	3.6E-05	1.3E-05	2.7E-07	5.5E-09	3.2E-05	6.0E-06	1.6E-07	1.3E-07	5.3E-15
	Best	6.5E-07	2.0E-05	1.3E-05	1.2E-07	6.5E-09	2.9E-06	7.9E-06	2.4E-07	1.0E-07	0.0E+00
$F_{27}$	Mean	1.4E-05	3.6E-08	2.4E-04	2.8E-07	1.3E-05	6.1E-07	7.1E-05	4.2E-06	5.8E-07	5.4E-14
	STD	2.5E-06	1.0E-08	2.4E-05	1.8E-08	2.0E-06	3.1E-08	1.2E-05	8.6E-07	1.4E-07	8.1E-15
	Best	3.6E-06	3.7E-09	3.9E-05	3.6E-08	1.9E-06	2.0E-08	8.9E-06	1.3E-06	9.3E-08	0.0E+00
$F_{28}$	Mean	6.4E-06	2.6E-04	2.3E-06	1.2E-04	3.4E-08	7.3E-08	1.2E-06	2.1E-06	2.9E-07	4.4E-13
	STD	1.7E-06	2.4E-05	5.3E-07	3.4E-05	7.7E-09	3.8E-09	2.9E-07	4.4E-07	5.2E-08	6.2E-14
	Best	2.1E-06	9.8E-06	4.5E-07	1.5E-05	2.6E-09	4.7E-09	2.3E-07	7.2E-07	2.3E-08	0.0E+00
$F_{29}$	Mean	1.9E-06	1.5E-06	1.7E-06	3.8E-07	4.3E-07	4.6E-06	8.5E-08	6.5E-07	2.9E-06	4.6E-11
	STD	4.4E-07	3.4E-07	4.2E-07	5.2E-08	1.1E-07	1.1E-06	1.6E-08	1.4E-07	6.7E-07	1.1E-11
	Best	6.0E-07	1.9E-07	3.0E-07	4.0E-08	5.5E-08	5.4E-07	8.1E-09	2.0E-07	2.7E-07	0.0E+00
$F_{30}$	Mean	5.1E-07	4.7E-07	4.0E-08	3.9E-04	2.4E-04	2.0E-08	1.1E-07	1.9E-07	1.1E-04	2.9E-14
	STD	1.2E-07	1.1E-07	5.4E-09	5.6E-05	4.6E-05	5.3E-09	8.5E-09	4.0E-08	7.3E-06	8.0E-15
	Best	1.8E-07	7.4E-08	6.9E-10	5.8E-05	4.0E-05	2.3E-09	2.1E-08	6.3E-08	1.2E-05	0.0E+00
$F_{31}$	Mean	7.4E-07	4.5E-07	5.7E-08	3.1E-07	1.3E-04	2.4E-06	3.2E-08	2.8E-07	1.6E-08	3.6E-14
	STD	1.7E-07	1.1E-07	1.3E-08	4.1E-08	2.9E-05	3.0E-07	4.7E-09	5.6E-08	1.1E-09	3.9E-15
	Best	2.6E-07	8.0E-08	9.8E-09	3.4E-08	1.3E-05	1.8E-07	1.4E-09	8.7E-08	6.7E-10	0.0E+00
$F_{32}$	Mean	2.0E-06	2.5E-04	2.2E-08	4.5E-05	2.6E-06	3.8E-07	4.1E-07	7.0E-07	3.6E-08	5.1E-14
	STD	4.9E-07	7.0E-05	4.4E-09	5.6E-06	3.2E-07	7.0E-08	4.1E-08	1.6E-07	6.4E-09	1.4E-14
	Best	6.8E-07	3.1E-06	3.5E-09	2.3E-06	4.4E-07	6.7E-08	4.2E-08	2.3E-07	4.2E-09	0.0E+00

**Table 8:** Execution times of the algorithms on the unimodal benchmark functions.

F	BCOA	GA	AL-SHADE	PSO	HAEA	AMRCSO	WOA	KOA	CBOA	PFO
F1	0.047	0.187	0.133	0.285	0.056	0.391	0.309	0.319	0.069	0.149
F2	0.265	0.333	0.176	0.106	0.231	0.041	0.334	0.260	0.308	0.152
F3	0.015	0.197	0.061	0.079	0.280	0.303	0.388	0.144	0.161	0.136
F4	0.037	0.106	0.105	0.186	0.336	0.286	0.139	0.336	0.326	0.199
F5	0.037	0.096	0.022	0.319	0.273	0.288	0.317	0.194	0.236	0.087
F6	0.044	0.235	0.121	0.261	0.230	0.232	0.136	0.032	0.186	0.096
F7	0.011	0.042	0.086	0.132	0.272	0.232	0.318	0.272	0.174	0.089
F8	0.024	0.294	0.052	0.081	0.210	0.078	0.285	0.190	0.165	0.126
F9	0.014	0.065	0.109	0.365	0.286	0.121	0.388	0.316	0.292	0.140
F10	0.033	0.193	0.076	0.136	0.240	0.087	0.346	0.308	0.293	0.172
F11	0.267	0.052	0.144	0.036	0.208	0.145	0.075	0.059	0.243	0.059
F12	0.029	0.245	0.038	0.384	0.203	0.317	0.051	0.205	0.206	0.117
F13	0.021	0.146	0.170	0.187	0.028	0.334	0.361	0.073	0.231	0.156
F14	0.023	0.296	0.187	0.061	0.368	0.107	0.034	0.231	0.161	0.272
F15	0.046	0.131	0.256	0.117	0.376	0.083	0.037	0.185	0.397	0.332
F16	0.015	0.217	0.118	0.313	0.271	0.162	0.056	0.304	0.120	0.185

**Table 9:** Execution times of the algorithms on the multimodal benchmark functions.

	BCOA	GA	AL-SHADE	PSO	HAEA	AMRCSO	WOA	KOA	CBOA	PFO
F17	0.011	0.078	0.072	0.248	0.352	0.095	0.138	0.315	0.389	0.183
F18	0.015	0.070	0.096	0.066	0.212	0.284	0.241	0.096	0.326	0.108
F19	0.035	0.372	0.101	0.134	0.206	0.272	0.383	0.129	0.371	0.045
F20	0.043	0.073	0.177	0.387	0.246	0.375	0.326	0.198	0.318	0.046
F21	0.323	0.108	0.129	0.020	0.350	0.249	0.177	0.162	0.182	0.280
F22	0.110	0.303	0.293	0.060	0.045	0.017	0.076	0.333	0.138	0.139
F23	0.030	0.078	0.055	0.028	0.041	0.086	0.040	0.245	0.279	0.157
F24	0.014	0.343	0.029	0.089	0.110	0.115	0.237	0.178	0.039	0.143
F25	0.015	0.040	0.147	0.058	0.341	0.363	0.392	0.325	0.316	0171
F26	0.025	0.215	0.162	0.196	0.166	0.263	0.121	0.073	0.202	0.113
F27	0.036	0.144	0.099	0.281	0.133	0.381	0.368	0.203	0.145	0.248
F28	0.039	0.222	0.116	0.129	0.299	0.097	0.284	0.347	0.070	0.130
F29	0.014	0.376	0.037	0.384	0.324	0.246	0.317	0.322	0.379	0.099
F30	0.254	0.085	0.191	0.238	0.197	0.219	0.310	0.324	0.015	0.261
F31	0.016	0.270	0.075	0.314	0.275	0.147	0.361	0.310	0.123	0.128
F32	0.076	0.376	0.048	0.166	0.297	0.230	0.376	0.317	0.202	0.123

**Table 10:** Wilcoxon Signed-Rank Test Results for Unimodal Benchmark Functions.

Algorithm	*p*-Value	Result
BCOA vs. GA	0.0004	+
BCOA vs. AL-SHADE	0.0078	+
BCOA vs. PSO	0.0002	+
BCOA vs. HAEA	0.0035	+
BCOA vs. AMRCSO	0.012	+
BCOA vs. WOA	0.0185	+
BCOA vs. KOA	0.065	0
BCOA vs. CBOA	0.0213	+
BCOA vs. PFO	0.031	+

**Table 11:** Wilcoxon Signed-Rank Test Results for Multimodal Benchmark Functions.

Algorithm	*p*-Value	Result
BCOA vs. GA	0.0025	+
BCOA vs. AL-SHADE	0.061	+
BCOA vs. PSO	0.031	+
BCOA vs. HAEA	0.054	0
BCOA vs. AMRCSO	0.0825	0
BCOA vs. WOA	0.0009	+
BCOA vs. KOA	0.11	0
BCOA vs. CBOA	0.0175	+
BCOA vs. PFO	0.0093	+

**Table 12:** Overall Win/Tie/Loss Statistics of BCOA Against Compared Algorithms.

Algorithm	Win (+)	Tie (=)	Loss (−)
BCOA vs. GA	2	0	0
BCOA vs. AL-SHADE	2	0	0
BCOA vs. PSO	2	0	0
BCOA vs. HAEA	1	1	0
BCOA vs. AMRCSO	1	1	0
BCOA vs. WOA	2	0	0
BCOA vs. KOA	0	2	0
BCOA vs. CBOA	2	0	0
BCOA vs. PFO	2	0	0

**Table 13:** Friedman Ranking Results on the 32 Benchmark Functions.

Function	BCOA	GA	AL-SHADE	PSO	HAEA	AMRCSO	WOA	KOA	CBOA	PFO
F1	1	6	2	4	8	5	5	9	10	3
F2	1	5	2	4	9	6	5	8	10	3
F3	1	7	2	5	8	6	4	9	10	3
F4	1	6	2	4	8	5	5	9	10	3
F5	1	7	3	4	9	6	5	8	10	2
F6	1	6	2	5	8	6	4	9	10	3
F7	2	5	3	4	8	6	5	9	10	1
F8	1	6	2	4	9	5	5	8	10	3
F9	1	5	2	4	9	6	5	8	10	3
F10	1	6	2	4	8	5	5	9	10	3
F11	1	5	2	5	8	6	4	9	10	3
F12	1	6	2	4	8	5	5	9	10	3
F13	1	7	3	4	9	6	5	8	10	2
F14	1	6	2	5	8	6	4	9	10	3
F15	2	5	3	4	8	6	5	9	10	1
F16	1	6	2	4	9	5	5	8	10	3
F17	1	6	2	5	8	6	4	9	10	3
F18	1	5	2	4	8	6	5	9	10	3
F19	1	6	2	4	9	5	5	8	10	3
F20	1	7	3	4	8	6	5	9	10	2
F21	1	6	2	5	8	6	4	9	10	3
F22	1	5	2	4	8	6	5	9	10	3
F23	1	6	2	4	9	5	5	8	10	3
F24	1	7	3	4	8	6	5	9	10	2
F25	1	6	2	5	8	6	4	9	10	3
F26	1	5	2	4	8	6	5	9	10	3
F27	1	6	2	4	9	5	5	8	10	3
F28	1	7	3	4	8	6	5	9	10	2
F29	1	6	2	5	8	6	4	9	10	3
F30	2	5	3	4	8	6	5	9	10	1
F31	1	6	2	4	9	5	5	8	10	3
F32	1	7	3	4	8	6	5	9	10	2

**Table 14:** Average Friedman Ranks of the Compared Algorithms.

Algorithm	Average Rank
BCOA	1.09
PSO	4.44
GA	5.94
AL-SHADE	2.25
PFO	2.75
WOA	4.75
AMRCSO	5.75
HAEA	8.00
KOA	8.72
CBOA	9.97

**Table 15:** Vargha–Delaney effect size (A) of BCOA against competing algorithms.

Algorithm	A Value	Effect
PSO	0.72	Large
GA	0.76	Large
AL-SHADE	0.61	Small
WOA	0.70	Medium
KOA	0.65	Small
AMRCSO	0.79	Large
HAEA	0.75	Large
CBOA	0.70	Medium
PFO	0.63	Small

**Table 16:** Ablation analysis of BCOA components over 32 benchmark functions.

Algorithm Variant	Average Rank
BCOA (Full)	1.0
BCOA without Reproduction/Inheritance	2.18
BCOA without Territorial Competition	2.96
BCOA without Adaptive Disturbance	3.74
BCOA without Angle-based Movement	4.62

**Table 17:** Problems, parameters, and their optimal values.

Problem	Parameters	Optimal Values
Pressure Vessel	Ts, Th, R, L	Ts = 0.81, Th = 0.44, R = 42.10, L = 176.64
Welded Beam	h, l, t, b	h = 0.21, l = 3.47, t = 9.04, b = 0.21
Spring Design	d, D, N	d = 0.05, D = 0.36, N = 11.29
Speed Reducer	b, m, z, l1, l2, d1, d2	b = 3.50, m = 0.70, z = 17.00, l1 = 7.30, l2 = 7.80, d1 = 3.35, d2 = 5.29
Edge Server Placement	X, A, C, D	X = 3.00, A = 120.00, C = 85.00, D = 12.40
Task Offloading Optimization	O, B, F, E, T	O = 0.70, B = 18.50, F = 2.40, E = 1.90, T = 0.85
UAV Deployment	P, R, E, Cov	P = 45.20, R = 120.00, E = 8.30, Cov = 92.50

**Table 18:** Reliability statistics of BCOA on engineering design problems.

Problem	Best	Mean	Std	Successful Runs	Reliability (%)
Pressure Vessel	5885.33	5887.12	2.36	29	96.7
Welded Beam	1.7248	1.7252	0.0004	30	100.0
Spring Design	0.01272	0.01275	0.00005	28	93.3
Speed Reducer	2994.81	2995.44	0.88	27	90.0
Edge Server Placement	1542.67	1545.92	3.74	28	93.3
Task Offloading	872.14	874.03	1.92	29	96.7
UAV Deployment	631.58	633.41	2.15	27	90.0

## Data Availability

Data available on request from the authors.

## Appendix A

### Numerical Example of the Quasi-Genetic Path Transition

This appendix provides a numerical example to illustrate the offspring angle generation process used in the quasi-genetic path transition mechanism described in Section 3.2. The example demonstrates the probabilistic parent selection process, the calculation of fitness-based weights, and the generation of offspring angles according to Equations (3)–(5).

Assume a population containing several candidate solutions. For simplicity, consider two individuals that may be selected as parents with the following fitness values:

$$f(X_{p})=2$$

$$f(X_{m})=5$$

**Step 1**: Parent selection using probabilistic strategy:

According to the fitness-based probabilistic selection mechanism, the selection probability of each individual is defined as

$$P_{i}=\frac{1/f(X_{i})}{\sum_{k=1}^{N}\left(1/f(X_{k})\right)}$$

Individuals with smaller objective function values, therefore, obtain higher probabilities of being selected. Based on this mechanism, assume that two individuals are selected as parents with the following migration angles:

$$\theta_{p}=60^{\circ }$$

$$\theta_{m}=40^{\circ }$$

**Step 2**: Compute the fitness-based weights:

According to the updated Equation (5), the weights are calculated using

$$w_{p}=\frac{1}{f(X_{p})+\varepsilon }$$

$$w_{m}=\frac{1}{f(X_{m})+\varepsilon }$$

Assume a small constant ε=0.01

Substituting the values:

$$w_{p}=\frac{1}{2+0.01}=0.4975$$

$$w_{m}=\frac{1}{5+0.01}=0.1996$$

**Step 3**: Compute the weighted mean migration angle:

The weighted mean of the parental angles is calculated as

$$\theta_{\mathrm{mean}}=\frac{w_{p}\theta_{p}+w_{m}\theta_{m}}{w_{p}+w_{m}}$$

$$\theta_{\mathrm{mean}}=\frac{0.4975\times 60+0.1996\times 40}{0.4975+0.1996}$$

$$\theta_{\mathrm{mean}}=\frac{29.85+7.98}{0.6971}\approx 54.28^{\circ }$$

**Step 4**: Compute the offspring angles:

Assume the following parameters:

$$\lambda_{1}=0.1$$

$$\lambda_{2}=0.15$$

$$\varepsilon_{1}=1^{\circ }$$

$$\varepsilon_{2}=-0.5^{\circ }$$

The difference between the parental angles is

$$\theta_{p}-\theta_{m}=20^{\circ }$$

Using Equations (3) and (4):

First offspring:

$$\theta_{c1}^{0}=\theta_{\mathrm{mean}}+\lambda_{1}(\theta_{p}-\theta_{m})+\varepsilon_{1}$$

$$\theta_{c1}^{0}=54.28+(0.1\times 20)+1=57.28^{\circ }$$

Second offspring:

$$\theta_{c2}^{0}=\theta_{\mathrm{mean}}-\lambda_{2}(\theta_{p}-\theta_{m})+\varepsilon_{2}$$

$$\theta_{c2}^{0}=54.28-(0.15\times 20)-0.5=50.78^{\circ }$$

This example illustrates how the proposed mechanism combines probabilistic parent selection, fitness-based weighting, and angular perturbations to generate diversified offspring migration angles. The weighted averaging promotes exploitation by guiding the search toward promising regions, while angular deviations and perturbations introduce diversity that enhances exploration. This balance between exploration and exploitation improves the algorithm’s ability to avoid premature convergence and maintain effective global search performance.
